# Adverse Childhood Experiences and Mortality at Old Age: A Longitudinal Study from the Japan Gerontological Evaluation Study

**DOI:** 10.1007/s40653-025-00732-y

**Published:** 2025-12-20

**Authors:** Iwao Chishima, Chie Koga, Kazushige Ide, Katsunori Kondo

**Affiliations:** 1 Futaba Clinic, Futaba-Cho, Utsunomiya, Tochigi Japan; 2 Institute for the Future of Human Society, Kyoto University, Kyoto, Japan; 3 Center for Preventive Medical Sciences, Chiba University, Chiba, Japan; 4 Institute for Health Economics and Policy, Association for Health Economics Research and Social Insurance and Welfare, Tokyo, Japan

**Keywords:** Adverse childhood experiences, ACE, Cumulative number of adverse childhood experiences, Parental loss, Parental mental illness, Physical abuse

## Abstract

Adverse childhood experiences (ACEs) are associated with undesirable adulthood health outcomes. However, studies investigating the effect of ACE components and cumulative number of ACEs on mortality in older adults are scarce. To examine the association between ACE components or cumulative number of ACEs and mortality in physically and cognitively independent older adults, separately by sex. We conducted a longitudinal study on 11,734 older adults aged ≥ 65 years who responded to the self-administered mail survey of the Japan Gerontological Evaluation Study in 2013. We adjusted for 20 confounders, including demographic and psychosocial factors. Modified Poisson regression models were used for calculating the risk ratio (RR), 95% confidence interval (CI), and p-value for mortality approximately 6 years later, separately by sex. A total of 1,498 (12.8%) participants died during the observation period. Parental mental illness (RR, 1.84; 95% CI: 1.08–3.12; p = 0.024) and physical abuse (RR, 1.60; 95% CI: 1.13–2.26; p = 0.008) in men and parental loss (RR, 1.24; 95% CI: 1.03–1.49; p = 0.021) or cumulative number of ACEs (RR, 1.21; 95% CI: 1.01–1.44; p = 0.035) in women were associated with higher RR for mortality. Men who had experienced physical abuse or parental mental illness and women who had lost their parents or experienced one ACE had a higher RR for mortality than those who had not. Further efforts to accumulate prospective studies and implement interventions are warranted.

**Supplementary Information:**

The online version contains supplementary material available at 10.1007/s40653-025-00732-y.

## Introduction

### Background

Adverse Childhood Experiences (ACEs) are broadly defined as potentially traumatic events that occur before the age of 18 and encompass a wide range of psychological, physical, and environmental stressors during childhood or adolescence. These experiences include direct forms of maltreatment, such as emotional abuse (e.g., verbal insults and humiliation), physical abuse, sexual abuse, and neglect (both physical and emotional). ACEs also extend to indicators of household dysfunction, such as parental separation or divorce, witnessing domestic violence (particularly violence directed toward the mother), living with family members who suffer from substance use disorders, alcohol dependence, or mental illnesses such as depression, as well as exposure to suicidal behavior, self-harm, or incarceration of a household member. (Felitti et al., 1998). Globally, 39% of adults had encountered ACEs (Kessler et al., 2010). ACEs cause neurodevelopmental deficits in childhood that impair interpersonal, emotional, and cognitive development, thereby leading to health-threatening behaviors, including alcohol use, smoking, and unhealthy lifestyles, which shorten life expectancy through physical or mental illness and physical or cognitive decline in adulthood (Amemiya et al., 2018; Amemiya et al., 2019; Anda et al., 2002; Bellis et al., 2015; Brown et al., 2010; Cheong et al., 2017; Dong et al., 2004; Felitti et al., 1998; Merrick et al., 2019; Rogers et al., 2021; Tani et al., 2020; Waehrer et al., 2020; Y. Wang et al., 2023a, 2023b). Moreover, at old age, ACEs have been associated with the development of dementia (Tani et al., 2020), decline in physical function (Amemiya et al., 2018), and even abuse (Review #1).

In a study conducted in the US in 2019, ACEs were associated with 15% of all-cause mortality (Grummitt et al., 2021). Currently, ACEs are social determinants of preventable mortality and a significant public health priority in adults. Several components of ACEs are associated with increased mortality (Chen et al., 2016; Hiyoshi et al., 2021; Larson & Halfon, 2013). Moreover, the number of ACEs is referred to as the cumulative number of ACEs; higher cumulative number of ACEs is associated with a higher risk of early mortality in adulthood (Bellis et al., 2015; Brown et al., 2009; Chen et al., 2016; Yu et al., 2022). However, most previous studies have included participants ranging from young to middle-aged adults. Few studies have specifically examined whether individual ACE components or the cumulative number of ACEs are associated with subsequent mortality risk among individuals who experienced ACEs and survived into an older age (Johnson et al., 2020). Typically, women are more affected by ACE components (Chen et al., 2016; Lee & Ryff, 2019; Smith et al., 2014) and cumulative number of ACEs (Chen et al., 2016) than men. This finding may be related to the fact that women have biological, psychological, and social characteristics that extend the effects of ACEs (Bale & Epperson, 2015; Chen et al., 2016; Kelly-Irving et al., 2013). Therefore, the association between ACE components or cumulative number of ACEs and mortality at old age should be evaluated separately for men and women.

Therefore, we included older adults living in various communities and had no decline in cognitive and physical function more than 50 years after experiencing ACEs. This study aimed to examine the association between ACE components or cumulative number of ACEs and risk of death after adjusting for confounders, separately for men and women.

## Methods

### Data Resource and Study Sample

**Fig. 1:**
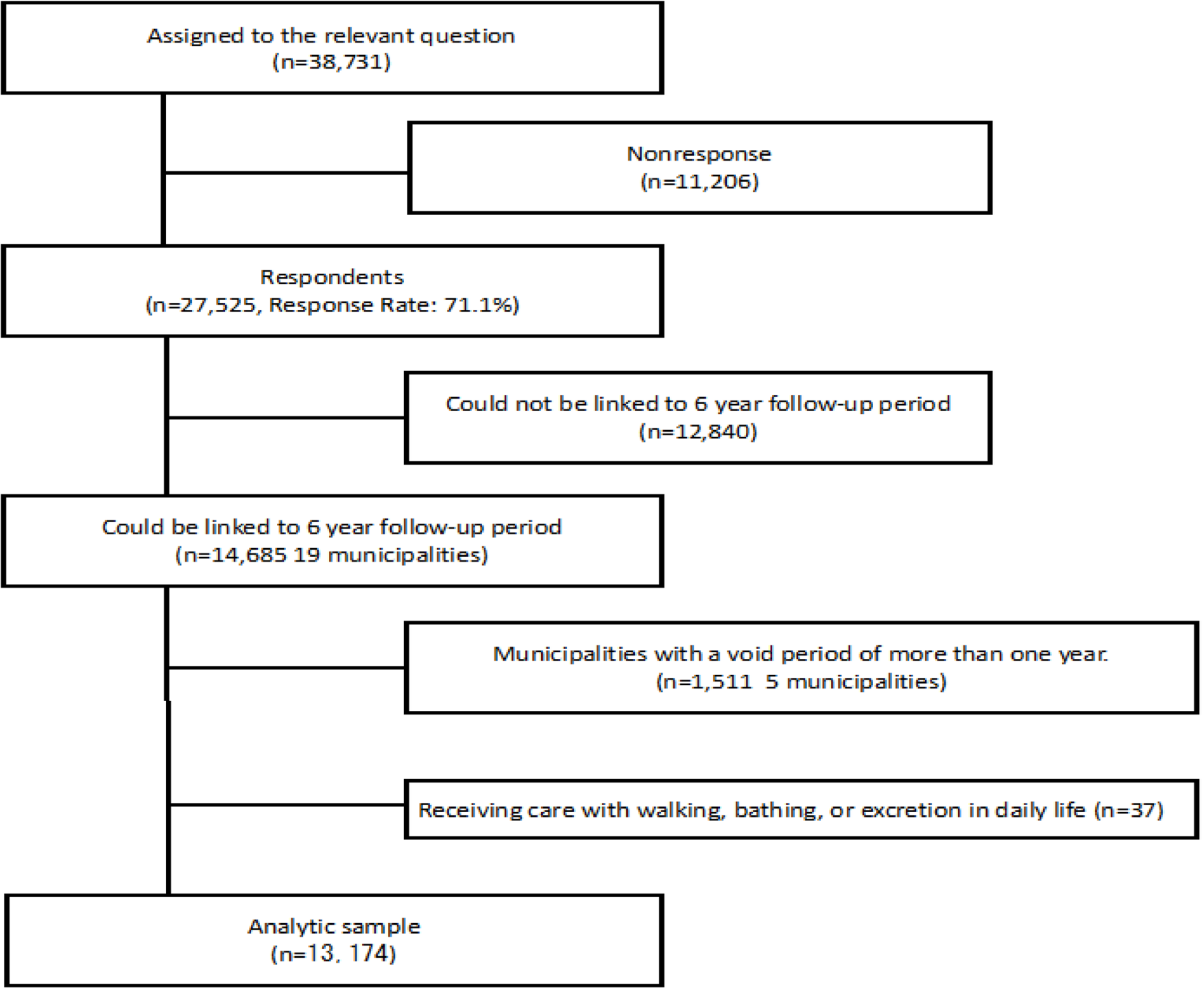
Flowchart of study participants

We used the data from the Japan Gerontological Evaluation Study (JAGES) (Anonymized for Review #2 and #3), a baseline survey of community-dwelling older adults in 24 municipalities across Japan conducted in 2013. Self-administered questionnaires were mailed to adults aged 65 years or older who were physically and cognitively independent, defined as individuals without functional disabilities and not certified as eligible for long-term care insurance benefits. One-fifth of the participants (n = 38,731) were randomly assigned to receive the ACE questionnaire, and 27,525 of them returned the completed questionnaire, resulting in a response rate of 71.1%. Among these respondents, 19 municipalities (n = 14,685) had mortality data available for linkage. We excluded 1,511 participants from five municipalities where mortality data were missing for more than one year, leaving 13,174 participants for analysis. Additionally, 29 individuals who reported receiving care or assistance with walking, bathing, or toileting in daily life were excluded. Consequently, the final analytical sample consisted of 13,174 participants (Fig. 1).

Participants were informed that participation in the study was voluntary, and that the completion and return of the questionnaire would be regarded as consent to participate. The study protocol was approved by the Ethics Committee of Chiba University (approval number: M10460).

### Definition of ACEs

The ACEs questionnaire used in this study was based on the original ACE questionnaire developed by Felitti et al. (Felitti et al., 1998). It included seven items assessing whether participants had experienced, by the age of 18, (1) loss of interpersonal relationships (parental bereavement or divorce), (2) family psychopathology (parental mental illness or domestic violence), or (3) abuse or neglect (physical abuse, psychological neglect, or psychological abuse) (parental mental illness, domestic violence), or abuse or neglect (physical abuse, psychological neglect, psychological abuse) (Amemiya et al., 2018; Matsuyama et al., 2016; Tani et al., 2020). Items related to child sexual abuse, parental substance use, and parental incarceration were excluded due to their low prevalence in Japan (Fujiwara, 2022). For each of the seven ACEs, we created binary variables indicating presence or absence, which we defined as individual ACE components. The cumulative number of ACEs was defined as the total number of components experienced before the age of 18. This cumulative score was categorized into three groups: 0, 1, and 2 or more ACEs. The categorization was based on the distribution of the data, as only 1.6% of participants reported experiencing three or more ACEs (Tani et al., 2020).

### Definition of Mortality

Mortality information was obtained through individual-level linkage between participant records and administrative death registries maintained by the local governments. The follow-up period for mortality surveillance began at the time of the baseline survey in 2013 and continued until the end of 2019, yielding an observation period of approximately six years. Mortality was defined as any death from any cause (all-cause mortality) occurring during this follow-up period. The date of death was confirmed through official records, ensuring accuracy and completeness of mortality ascertainment.

### Covariates

Covariates were assessed using a self-administered questionnaire. Age was treated as a continuous variable. Childhood environmental factors included educational history, economic hardship during childhood (Amemiya et al., 2018), and height (Fujiwara et al., 2014; Silventoinen, 2003). Education was categorized into ≤ 9, 10–12, or ≥ 13 years of schooling. Economic hardship was assessed as a binary variable (yes or no). Height was used as a proxy for nutritional status and categorized into five groups by sex (men, < 155, 155–159.9, 160–164.9, 165–169.9, or ≥ 170 cm; women, < 145, 145–149.9, 150–154.9, 155–159.9, or ≥ 160 cm) (Fujiwara et al., 2014). Socioeconomic status in adulthood included equivalized income (< 1.99, 2–3.99, or 4 million yen) and employment status (currently employed, not currently employed, or never worked) (Tani et al., 2021). Health behaviors included smoking and alcohol use, categorized as current, quit, or never. The presence of chronic diseases under treatment included hypertension, diabetes, heart disease, stroke, lung disease, or cancer (Tani et al., 2021). Depression was measured using the Geriatric Depression Scale (Yesavage et al., 1982) and categorized into < 4, 5–9, or ≥ 10 points. Social relationships included social associations in adulthood (sports and participation in hobby groups), frequency of going out (more than or less than once a week), marital status (married [including common-law], widowed, separated, never married, or others), frequency of contact with friends and acquaintances (more than or less than once a week), number of contacts with friends and acquaintances (more than or less than three individuals) (Ashida et al., 2023).

### Statistical Analysis

We first described the baseline characteristics of the study participants, as well as the distribution of ACE components and the cumulative number of ACEs, stratified by sex. We then compared the characteristics of participants according to survival status.

Because the proportional hazards assumption was violated, as indicated by Kaplan–Meier plots, we employed modified Poisson regression with robust variance to estimate risk ratios (RRs), 95% confidence intervals (CIs), and p-values for mortality (Lee et al., 2009; McNutt et al., 2003; Zhang & Yu, 1998). Logistic regression was not used, as it may overestimate risk when event rates exceed 10% (Zhang & Yu, 1998). To ensure consistency in follow-up periods across municipalities, we aligned observation intervals before modeling.

We calculated RRs for mortality associated with each ACE component (compared with no exposure to that component), and RRs for cumulative ACE exposure (1 or ≥ 2 ACEs) compared with no ACEs, stratified by sex. Following previous studies (Tani et al., 2021), we included sociodemographic, socioeconomic, disease-related, health behavior, and social relationship variables across multiple models.

Covariates were entered into the models stepwise, in line with a life-course epidemiological framework and theoretical considerations of confounding. The crude model included only ACEs. Model 1 adjusted for age and childhood environmental factors (e.g., economic hardship, height, educational attainment), which may influence both ACE exposure and later health outcomes. Model 2 further adjusted for adulthood socioeconomic and health-related variables (e.g., income, employment status, health behaviors, and chronic conditions), which may mediate or confound the relationship between ACEs and mortality. Finally, Model 3 included adult social relationships variables (e.g., social participation, frequency of contact with friends), which could either buffer or amplify the long-term effects of ACEs on mortality.

This sequential modeling approach allowed us to examine the extent to which the association between ACEs and mortality remained independent of, or was explained by, life-course covariates.

We conducted three sensitivity analyses. First, because the association between age and mortality is nonlinear in Japan (Ministry of Health, 2021), we included a quadratic term for age in the model. Second, since including ACE components that were not associated with elevated mortality risk may obscure the association, we recalculated the cumulative ACE score using only components statistically associated with higher mortality and re-examined their effect in later life. Third, as our ACE questionnaire was adapted from the original instrument by Felitti et al. (Felitti et al., 1998) and modified for the Japanese adults (Amemiya et al., 2018; Matsuyama et al., 2016; Tani et al., 2020), we regrouped the ACEs into three conceptual domains: loss of interpersonal relationships, family psychopathology, and abuse or neglect. All analyses were conducted separately for men and women. We assumed that missing data occurred at random and addressed this using multiple imputation (Rubin, 1996). A total of 20 imputed datasets were generated using the multivariate normal imputation method in Stata. All analyses were performed using Stata version 17 (StataCorp LLC, College Station, TX, USA).

## Results

### Participant Characteristics

A total of 1,498 (12.8%) mortality events were observed during the observation period. Of the participants, 950 (17.6%) and 568 (8.6%) were men and women, respectively. The baseline characteristics of the participants are presented in Table 1. The proportion of women was 54%. A higher proportion of older adults aged 65–74 years was noted than those aged ≥ 75 years. Parental loss (22.7%) was the most common ACE component, followed by psychological neglect (12.5%). Parental separation (1.9%), parental mental illness (0.7%), and physical abuse (1.4%) were the ACE components that participants were less likely to have experienced. A greater proportion of men than women had encountered ACEs. Of the participants, 37% had experienced one or more ACEs, and 7.9% had experienced two or more ACEs. Additionally, of the participants, 45% experienced childhood deprivation, 35% had < 9 years of education, and men were more likely to have a higher education level. More than 40% of the participants had an equivalized income of < 2 million yen at older age. Manual labor constituted 63% of the longest occupation. Smoking, heart disease, diabetes, and stroke were more common in men. Of the participants, 60%–70% had a spouse. women met with friends more frequently than men; however, social participation was comparable between men and women. Men were more likely to be employed than women.

**Table 1:** Characteristics of the study participants (*n* = 13,174)

Characterics		Total		Male		Female	
		n	%	n	%	n	%
Age	Continuous Variable	73.8	0.1	73.6	0.1	74.0	0.1
Adverse childhood experiences							
Interpersonal loss							
Parental death	Yes	3,155	24	1,470	32,8	1,685	23,8
	No	9,332	71.0	4,253	70.3	5,079	71.7
	Missing	650	5.0	328	5.4	322	4.5
Parental divorce	Yes	267	2.0	144	2.4	123	1.7
	No	11,857	90.3	5,410	89.4	6,447	91.0
	Missing	1,013	7.7	497	8.2	516	7.3
Family psychopathology							
Parental mental illness	Yes	92	0.7	56	0.9	36	0.5
	No	12,051	91.7	5,511	91.1	6,540	92.3
	Missing	994	7.6	484	8.0	510	7.2
Family violence	Yes	507	3.9	292	4.8	215	3.0
	No	11,661	88.8	5,285	87.3	6,376	90.0
	Missing	969	7.4	474	7.8	495	7.0
Abuse and neglect							
Physical abuse	Yes	187	1.4	124	2.1	63	0.9
	No	12,015	91.5	5,469	90.4	6,546	92.4
	Missing	935	7.1	458	7.6	477	6.8
Psychological neglect	Yes	1,547	11.8	829	13.7	718	10.1
	No	10,788	82.1	4,787	79.1	6,001	84.7
	Missing	802	6.1	435	7.2	367	5.2
Psychological abuse	Yes	712	5.4	363	6.0	349	4.9
	No	11,511	87.6	5,225	86.4	6,286	88.7
	Missing	914	7.0	463	7.7	451	6.4
Childhood other environment							
Number of adverse childhood experiences	0	7,365	56.1	3,198	52.9	4,167	58.8
	1	3,448	26.3	1,676	27.7	1,772	25.0
	2 +	921	7.0	512	8.5	409	5.8
	Missing	1,403	10.7	665	11.0	738	10.4
Economic hardship	Yes	5,823	44.3	3,101	51.3	2,722	38.4
	No	6,650	50.6	2,622	43.3	4,028	56.8
	Missing	664	5.1	328	5.4	336	4.7
Height	Short			399	6.6	769	10.9
	middle short			934	15.4	1,885	26.6
	Middle			1,832	30.3	2,458	34.7
	middle tall			1,679	27.8	1,347	19.0
	Tall			1,097	18.1	409	5.8
	Missing			110	1.8	218	3.1
Education, years	Low (≤ 9)	5,181	39.4	2,177	36.0	3,004	42.4
	Middle (10–12)	4,987	38.0	2,200	36.4	2,787	39.3
	High (≥ 13)	2,681	20.4	1,581	26.1	1,100	15.5
	Other/missing	288	2.2	93	1.5	195	2.8
Adult socioeconomical state							
Annual income, million yen	Low (< 2.00)	5516	42.0	2,604	43.0	2,912	41.1
	Middle (2.00–3.99)	4060	30.9	2,095	34.6	1,965	27.7
	High (≥ 4.00)	1068	8.1	546	9.0	522	7.4
	Missing	2493	19.0	806	13.3	1,687	23.8
Longest occupation	Non-manual	2,829	21.5	2,127	35.2	702	9.9
	Manual	8,111	61.7	3,402	56.2	4,709	66.5
	No occupation	631	4.8	30	0.5	601	8.5
	Missing	1,566	11.9	492	8.1	1,074	15.2
Health behaviors							
Smoking	Smoking	1,343	10.2	1,111	18.4	232	3.3
	Smoker/ex-smoker	1,998	15.2	1,850	30.6	148	2.1
	Nonsmoker	9,571	72.9	2,997	49.5	6,574	92.8
	Missing	225	1.7	93	1.5	132	1.9
Drinking	Drinking	4,633	35.3	3,480	57.5	1,153	16.3
	Drinker/ex-drinker	607	4.6	495	8.2	112	1.6
	Nondrinker	7,701	58.6	1,985	32.8	5,716	80.7
	Missing	196	1.5	91	1.5	105	1.5
Health status							
BMI	Underweight (< 18.5)	858	6.5	302	5.0	556	7.9
	Normal (18.5–24.9)	8,825	67.2	4,153	68.6	4,672	65.9
	Overweight (25.0–29.9)	2,514	19.1	1,261	20.8	1,253	17.7
	Obesity (≥ 30.0)	288	2.2	101	1.7	187	2.6
	Missing	652	5.0	234	3.9	418	5.9
Disease	Hypertension	5,738	43.7	3,086	51.0	3,464	48.9
	Diabetes mellitus	1,720	13.1	1,002	16.6	718	10.1
	Stroke	397	3.0	254	4.2	143	2.0
	Heart disease	1,405	10.7	813	13.4	592	8.4
	Lung disease	636	4.8	355	5.9	281	4.0
	Cancer	458	3.5	269	4.5	189	2.7
Depressive symptoms	No depression (GDS < 5)	8,124	61.8	3,862	63.8	4,262	60.2
	Moderate depression (5 ≤ GDS < 10)	2,114	16.1	1,060	17.5	1,054	14.9
	Depression (10 ≤ GDS)	683	5.2	368	6.1	315	4.5
	Missing	2,216	16.9	761	12.6	1,455	20.5
Adult social relationships							
Marital status	Married	9,290	70.7	5,146	85.0	4,144	58.5
	Widowed	2,699	20.6	453	7.5	2,246	31.7
	Divorced/unmarried/other	839	6.4	356	5.9	483	6.8
	Missing	309	2.4	96	1.6	213	3.0
Frequency of meeting friends	≥ 1/week	6,343	48.3	2,433	40.2	3,910	55.2
	≥ 1/month	2,806	21.4	1,314	21.7	1,492	21.1
	Rarely	3,316	25.2	2,033	33.6	1,283	18.1
	Missing	672	5.1	271	4.5	401	5.7
Social participation	Yes	2,853	21.7	1,391	23.0	1,462	20.6
	No	8,800	67.0	4,071	67.3	4,729	66.7
	Missing	1,484	11.3	589	9.7	895	12.6
Employment status	Working	2,749	20.9	1,653	27.3	1,096	15.5
	Retired	7,801	59.4	3,839	63.4	3,962	55.9
	Never worked	1,464	11.1	236	3.9	1,228	17.3
	Missing	1,123	8.6	323	5.3	800	11.3

Height: short, < 155 cm; medium short, 155–159.9 cm; medium, 160–164.9 cm; medium tall, 165–169.9 cm; and tall, ≥ 170 cm for men and short, < 145 cm; medium short, 145–149.9 cm; medium, 150–154.9 cm; medium tall, 155–159.9 cm; and tall, ≥ 160 cm for women.

Age is listed as mean and standard deviation. *GDS* Geriatric Depression Scale.

Controlled for age, childhood economical hardship, height, education, annual income, longest occupation, smoing, drinking, BMI, Disease, depressive symptom, martial status, frequency of meeting friends, social participation, employment status.

*ACEs* Adverse Childhood Experiences

The ACE components and cumulative number of ACEs are shown in Table 2. More than 50% of men and women had experienced parental loss. For the cumulative number of ACEs = 1, 26% and 21% of men and women, respectively, had experienced psychological neglect. A cumulative number of ACEs = 2 + had a higher percentage of participants who encountered ACEs than a cumulative number of ACEs = 1, except for parental loss. Compared with the cumulative number of ACEs = 1, the cumulative number of ACEs = 2 + had a particularly high proportion of participants who experienced psychological abuse or psychological neglect.

**Table 2:** ACE components and cumulative number of ACEs by sex (*n* = 13,174)

Men				
	ACEs = 1(n = 1,676)		ACEs = 2 + (n = 512)	
	n	%	n	%
Parental loss	959	57.2	302	59.0
Parental divorce	46	2.7	77	15.0
Parental mental illness	12	0.7	33	6.5
Family violence	95	5.7	165	32.2
Physical abuse	11	0.7	98	19.1
Psychological neglect	431	25.7	366	71.5
Psychological abuse	122	7.3	208	40.6
Total	1676	100.0	1249	243.9
**Women**				
	ACEs = 1(n = 1,772)		ACEs = 2 + (n = 409)	
	n	%	n	%
Parental loss	1,147	64.7	253	61.9
Parental divorce	53	3.0	45	11.0
Parental mental illness	9	0.5	22	5.4
Family violence	84	4.7	106	25.9
Physical abuse	5	0.3	48	1.7
Psychological neglect	368	20.8	306	74.8
Psychological abuse	106	6.0	198	48.4
Total	1,772	100.0	978	229.1

*ACEs* Adverse Childhood Experiences

Age at study enrollment and age at mortality for each ACE component and cumulative number of ACEs are shown in Supplementary Table 1. Regardless of ACE components and cumulative number of ACEs, the mortality group was approximately 4–5 years younger for men and 5–6 years younger for women than the survival group.

### Association between ACEs and RR for Mortality

The association between ACE components and mortality is shown in Table 3. For men, physical abuse and parental mental illness were associated with higher RR for mortality in the crude model and models 1–3 than never having experienced them. For women, parental loss was associated with higher RR for mortality in the crude model and models 1–3 than never having experienced it. Psychological abuse was associated with lower RR for mortality for men and women in models 2–3 than never having experienced it.

**Table 3:** Association between ACE components and risk of mortality (modified Poisson regression model, n = 11,734)

Men																
	Crude				Model1				Model2				Model3			
n = 5,386	IRR	95%CI		p	IRR	95%CI		p	IRR	95%CI		p	IRR	95%CI		p
Parental loss	1.26	1.11	1.42	< 0.001	1.09	0.96	1.23	0.187	1.06	0.94	1.20	0.358	1.07	0.94	1.21	0.303
Parental divorce	0.74	0.49	1.11	0.146	0.78	0.52	1.18	0.239	0.75	0.50	1.12	0.161	0.77	0.51	1.15	0.194
Parental mental illness	**1.85**	**1.24**	**2.78**	**0.003**	**1.93**	**1.29**	**2.88**	**0.001**	**1.86**	**1.22**	**2.84**	**0.004**	**1.93**	**1.27**	**2.95**	**0.002**
Family violence	0.76	0.56	1.03	0.079	0.80	0.59	1.08	0.150	0.79	0.59	1.07	0.125	0.82	0.61	1.09	0.172
Physical abuse	**1.62**	**1.14**	**2.32**	**0.008**	**1.61**	**1.13**	**2.29**	**0.008**	**1.55**	**1.11**	**2.16**	**0.009**	**1.51**	**1.09**	**2.09**	**0.013**
Psychological neglect	1.14	0.97	1.35	0.110	1.11	0.95	1.31	0.197	0.98	0.83	1.15	0.777	0.97	0.82	1.13	0.668
Psychological abuse	0.82	0.63	1.07	0.142	0.82	0.62	1.07	0.14	**0.74**	**0.57**	**0.97**	**0.027**	**0.74**	**0.57**	**0.96**	**0.025**
**Women**																
	Crude				Model1				Model2				Model3			
n = 6,348	IRR	95%CI		p	IRR	95%CI		p	IRR	95%CI		p	IRR	95%CI		p
Parental loss	**1.49**	**1.25**	**1.77**	** < 0.001**	**1.22**	**1.02**	**1.44**	**0.027**	**1.20**	**1.01**	**1.43**	**0.037**	**1.20**	**1.01**	**1.43**	**0.037**
Parental divorce	1.15	0.64	2.06	0.632	1.37	0.76	2.47	0.295	1.34	0.75	2.38	0.326	1.32	0.73	2.36	0.356
Parental mental illness	0.95	0.32	2.81	0.925	0.76	0.29	2.00	0.573	0.77	0.30	1.99	0.591	0.86	0.32	2.27	0.755
Family violence	1.15	0.73	1.83	0.550	1.43	0.91	2.26	0.125	1.38	0.89	2.13	0.152	1.44	0.93	2.24	0.101
Physical abuse	0.67	0.21	2.11	0.494	0.81	0.28	2.37	0.703	0.78	0.28	2.19	0.642	0.78	0.27	2.26	0.651
Psychological neglect	1.02	0.76	1.35	0.917	0.91	0.69	1.20	0.499	0.90	0.68	1.20	0.484	0.90	0.68	1.19	0.460
Psychological abuse	0.70	0.44	1.13	0.146	0.66	0.42	1.05	0.081	**0.53**	**0.33**	**0.87**	**0.013**	**0.51**	**0.31**	**0.85**	**0.009**

Controlled for age, childhood economical hardship, height, education, equivalized income, longest occupation, smoing, drinking, Disease, depressive symptom, martial status, frequency of meeting friends, social participation, employment status.

Crude includes ACEs components

Model 1 Includes Crude Model and age and adverse childhood experiences (economic status in childhood, height, educational history) and socioeconomic status in old age (equivalized income, employment status).

Model 2 includes Model 2 and health status in old age (hypertension, diabetes, dyslipidemia, heart disease, respiratory disease, cancer) and lifestyle (smoking and alcohol).

Model 3 includes Model 3 and social relationships in old age (social participation, frequency of interaction with friends, employment status, marital status).

*ACEs* Adverse Childhood Experiences, *IRR* Incidence Rate Ratio, *CI* Confidence Interval

The association between the cumulative number of ACEs and mortality is presented in Table 4. For women, a cumulative number of ACEs = 1 was associated with a higher RR for mortality than a cumulative number of ACEs = 0 in the crude model and models 1–3. A cumulative number of ACEs = 2 + was not associated with a higher RR for mortality than a cumulative number of ACEs = 0 (Table 4).

**Table 4:** Association between cumulative number of ACEs and risk of death stratified by sex (modified Poisson regression model, *n* = 11,734)

Men																
Number of ACEs	Crude				Model1				Model2				Model3			
	IRR	95%CI		p	IRR	95%CI		p	IRR	95%CI		p	IRR	95%CI		p
0	Ref.	Ref.		Ref.	Ref.	Ref.		Ref.	Ref.	Ref.		Ref.	Ref.	Ref.		Ref.
1	1.15	1.01	1.31	0.029	1.06	0.93	1.20	0.375	1.00	0.89	1.13	0.966	1.00	0.89	1.13	0.966
2+	1.22	1.01	1.47	0.037	1.11	0.91	1.35	0.291	0.92	0.76	1.12	0.424	0.93	0.77	1.13	0.466
Women																
Number of ACEs	Crude				Model1				Model2				Model3			
	IRR	95%CI		p	IRR	95%CI		p	IRR	95%CI		p	IRR	95%CI		p
0	Ref.	Ref.		Ref.	Ref.	Ref.		Ref.	Ref.	Ref.		Ref.	Ref.	Ref.		Ref.
1	1.44	1.21	1.72	<0.001	1.22	1.03	1.45	0.021	1.19	1.01	1.42	0.043	1.20	1.01	1.42	0.040
2+	1.06	0.75	1.50	0.734	0.92	0.65	1.31	0.637	0.81	0.55	1.18	0.267	0.80	0.55	1.17	0.245

Controlled for age, childhood economical hardship, height, education, equivalized income, longest occupation, smoing, drinking, Disease, depressive symptom, martial status, frequency of meeting friends, social participation, employment status.

Crude includes cumulative ACEs score

Model 1 Includes Crude Model and age and adverse childhood experiences (economic status in childhood, height, educational history) and socioeconomic status in old age (equivalized income, employment status).

Model 2 includes Model 2 and health status in old age (hypertension, diabetes, dyslipidemia, heart disease, respiratory disease, cancer) and lifestyle (smoking and alcohol).

Model 3 includes Model 3 and social relationships in old age (social participation, frequency of interaction with friends, employment status, marital status).

*ACEs* Adverse Childhood Experiences, *IRR* Incidence Rate Ratio, *CI* Confidence Interval

### Sensitivity Analysis

#### Changing the Age to a Squared Term

The association between the number of deaths by age and age at death is not linear but an upward convex parabola (Ministry of Health, 2021). To address the underestimation of the effects of ACEs with aging, we developed a squared term for age (continuous value) and entered the modified Poisson analysis. The association between ACE components and mortality after converting age into a squared term is shown in Supplementary Table 2. For men, parental mental illness and physical abuse were associated with a higher RR for mortality in the crude model and models 1–3 than never having experienced them. For women, parental loss was associated with a higher RR for mortality in the crude model and models 1–3 than never having experienced it. For men and women, psychological abuse was associated with a lower RR for mortality in models 2–3 than never having experienced it.

The association between cumulative number of ACEs and RR for mortality after converting age into a squared term is presented in Supplementary Table 3. For men, neither a cumulative number of ACEs of 1 nor 2 + was associated with a higher RR for mortality than a cumulative number of ACEs = 0. For women, a cumulative number of ACEs = 1 was associated with a higher RR for mortality than a cumulative number of ACEs = 0 in the crude model and models 1–3. Conversely, a cumulative number of ACEs = 2 + was not associated with a higher RR for mortality than a cumulative number of ACEs = 0.

#### Modifying the Cumulative Number of ACEs

As noted in the Methods section, we developed a modified cumulative number of ACEs. As parental loss, parental mental illness, and physical abuse were associated with higher RRs for mortality (Table 3), a modified cumulative number of ACEs was developed using only these three ACEs and analyzed using a Poisson regression model. The association between the modified cumulative number of ACEs and RR for mortality is presented in Supplementary Table 4. For men, a cumulative number of ACEs = 2 + was associated with a higher RR for mortality than a cumulative number of ACEs = 0 in the crude model and models 1–3. For women, a cumulative number of ACEs = 1 was associated with a higher RR for mortality than a cumulative number of ACEs = 0 in models 1–2 but not in models 2–3.

#### Reclassifying ACEs into Major Categories

As noted in the Methods section, we reclassified ACEs into the following broad categories: loss of interpersonal associations, family psychopathology, and abuse and neglect. For women, interpersonal associations were associated with a high RR for mortality in the crude model and models 1–3, and abuse and neglect were associated with a low RR for mortality only in model 3. For men, no association was noted between ACEs and a high RR for mortality (Supplementary Table 5).

## Discussion

### Summary of Results

We examined the individual components and cumulative number of ACEs and their associations with mortality risk among older adults, separately by sex. First, men who had experienced physical abuse or parental mental illness during childhood had a higher RR of mortality in old age than those who had not. Second, women who had experienced parental loss in childhood similarly showed a higher RR of mortality. Third, both men and women who had experienced psychological abuse in childhood demonstrated a lower RR of mortality in old age. Fourth, women with a cumulative ACE score of 1 had a significantly higher RR of mortality than those with a score of 0. This pattern was not observed in men. We found that among older adults who had experienced ACEs but were not certified for long-term care, both individual ACE components and cumulative exposure were associated with increased mortality risk over approximately six years, even after adjusting for covariates. While our findings are partially consistent with previous studies showing that childhood adversity is associated with higher mortality risk in old age (de Souza et al., 2022; Smith et al., 2014), research specifically focusing on older populations remains limited (Johnson et al., 2020). In contrast, our findings diverge from earlier studies that reported a dose–response relationship between higher ACE exposure and increased mortality risk in adulthood (Chen et al., 2016; D'Arcy-Bewick et al., 2023; Johnson et al., 2020).

### Strengths of this Study

Our study has two major strengths. First, the sample was restricted to participants aged 65 years and older, allowing us to demonstrate that the effects of ACEs persist into old age, even among those who avoided premature death and functional decline. Second, we analyzed sex-specific associations, revealing that the impact of ACEs on mortality may differ between men and women. Despite evidence suggesting gendered pathways in ACE-related health outcomes, few studies have separately examined mortality risk in older men and women. Association between ACEs and mortality after ≥ 65 years.

#### Parental Mental Illness in Men

Men who experienced parental mental illness during childhood exhibited higher mortality risk. Two mechanisms may underlie this association. First, parental mental illness affects children’s development through both direct (e.g., genetic predisposition, in utero stress, and parental modeling) and indirect (e.g., poverty, family dysfunction) pathways (Manning & Gregoire, 2009). Previous studies have linked such exposure to reduced preventive healthcare use (e.g., vaccinations) (Bente Kjær et al., 2018; Davidsen et al., 2021), increased physical illness (Renneberg et al., 2023), more frequent medical visits (Heuckendorff et al., 2021; Lyngsøe et al., 2019), overlapping with other ACEs (Y.-X. Wang et al., 2023a, 2023b), and elevated childhood mortality (Chen et al., 2010). Second, adolescent boys may be more vulnerable to these effects due to lower self-efficacy and greater difficulty seeking support in unstable families (Cavanagh et al., 2008; Giordano et al., 2006). Parental mental illness during this period can negatively affect adolescent men. For example, parental alcoholism causes adolescent men to experience more stress than adolescent women (Shera & Sher, 2017). Studies have shown that boys exposed to maternal depression or parental rejection suffer more adverse outcomes, including mental health problems and maladaptive behaviors (Shera & Sher, 2017). These adverse effects—especially among men—may persist into old age, contributing to elevated mortality risk later in life.

#### Physical Abuse and Mortality in Men

In our study, Childhood physical abuse was associated with elevated mortality risk in men. Prior research has linked physical abuse to increased mortality in both sexes (D'Arcy-Bewick et al., 2023; Rogers et al., 2021), though more severe consequences have been documented in women.

(Evans et al., 2013; Y.-X. Wang et al., 2023a, 2023b). In our sample, however, physical abuse was more commonly reported by men. Given the post-war context in which many participants were raised　(Peter, n.d.), the severity of physical abuse may have been greater among men in our study. Moreover, the number of women who experienced physical abuse during childhood may have been too small to yield a statistically significant difference.

#### Parental Loss and Mortality in Women

In our study, parental loss was associated with a higher RR for mortality in women. This finding is supported by substantial evidence indicating that parental loss is linked to increased mortality risk in adulthood, regardless of sex (Guldin et al., 2015; Hiyoshi et al., 2021; Larson & Halfon, 2013; Martin et al., 2005; Remes et al., 2011). Notably, in our cohort, more than one-fifth of participants aged ≥ 65 years (born before 1948) had experienced parental loss during childhood. This high prevalence may be largely attributable to the widespread mortality and familial disruption caused by the Pacific War. Parental death during or after the war often resulted in girls being deprived of food rations and medical care, having reduced access to health and welfare services, and receiving less social support (Johnson et al., 2020; Kelly-Irving et al., 2013; McKay, 1998; Under-Secretary-General, 2003). These gender-based disparities in postwar support systems may have contributed to elevated mortality risk among women. Furthermore, prior research suggests that men are more strongly affected by parental loss during early adulthood, whereas women are more affected by such loss during childhood (Smith et al., 2014). Social factors such as income, education, and family support have been shown to mediate the impact of parental loss on long-term health outcomes. However, the mediating effect of these factors appears to be weaker in women (Hailey Maier & Lachman, 2000), and occupational satisfaction and achievement may offer less protection against mortality risk for women than for men (Martin et al., 2005). These disparities may help explain why women face greater difficulty in developing effective coping mechanisms in the context of socioeconomic inequality.

#### Psychological Abuse and Mortality in Men and Women

Childhood psychological abuse was associated with a lower RR for mortality in both men and women in Models 3–4 and 2–4, respectively. This contrasts with previous studies reporting that psychological abuse in childhood is linked to increased risks of physical and mental illness (Dye, 2020; Sheikh, 2018, 2021) and higher mortality in adulthood (Chen et al., 2016; D'Arcy-Bewick et al., 2023). The participants in our study were born before 1947, and most spent their childhood during or shortly after the Pacific War. Many were exposed to uniquely harsh conditions such as forced labor, mass evacuations, wartime mobilization, or life as war orphans (Moore & Piel; Peter n.d.; Piel, 2016; Tamanoi, 2020). In such extreme circumstances, verbal abuse or emotional neglect from parents may have been perceived as relatively minor and thus less psychologically damaging. Moreover, children who were highly vulnerable to ACEs may have been excluded from the present study because they developed physical or mental impairments or died prematurely before reaching older age. Consequently, our sample may have consisted primarily of individuals who were more resilient to psychological adversity. In addition, those who reported no history of verbal insults or emotional harm from their parents may have experienced more stable family environments, characterized by the absence of parental loss or separation and the presence of psychological and social support.

#### Cumulative Number of ACEs and Mortality in Men

In our study, the cumulative number of ACEs was not associated with an increased RR of mortality among men. We propose two possible explanations for this finding. First, a substantial number of men may not have met the inclusion criteria due to death or physical or cognitive decline before reaching the age of 65 (Brown et al., 2009). Second, the inclusion of ACEs that were not individually associated with mortality risk may have diluted the overall association. To address this, we recalculated the cumulative number of ACEs using only those components that were significantly associated with mortality—namely, parental separation, parental mental illness, and physical abuse—and found that having two or more of these ACEs (2 +) was associated with a higher RR of mortality (Supplementary Table 4). This finding aligns with previous studies reporting that a higher cumulative number of ACEs is associated with an increased risk of death (Brown et al., 2009; Yu et al., 2022).

#### Cumulative Number of ACEs and Mortality In Women

Our results differ from those of previous studies (Johnson et al., 2020; Kelly-Irving et al., 2013), as we did not observe a higher RR of mortality among women with a cumulative number of ACEs of 2 or more compared to those with no ACEs. One possible explanation is that relatively healthy older adults were overrepresented in our sample, as individuals who had experienced ACEs may have been excluded due to physical or cognitive decline or premature death prior to reaching old age (Smith et al., 2014). The elevated RR for mortality could also be partially explained by age, as there was a difference of approximately 5–6 years between those who died and those who survived (Supplementary Table 1). However, to address this potential bias, we treated age as a continuous variable and conducted a sensitivity analysis including a quadratic term, which did not alter the results (Supplementary Table 3). Furthermore, 65% of the women with a cumulative ACE score of 1 had experienced parental loss during childhood. Therefore, it is possible that the observed effect in this group primarily reflects the impact of parental loss.

### Cultural and Historical Background

The interpretation of our findings should be situated within the unique cultural and historical context of the study population. Most participants in this study were born before or during the Pacific War and spent their formative years in postwar Japan—a period characterized by poverty, family separation, food shortages, and widespread adversity (Moore & Piel n.d.; Peter; Piel, 2016; Tamanoi, 2020). Traditional Japanese cultural values, particularly those rooted in the “culture of shame” and the ideal of “endurance” (Lebra, 1976), may have influenced how children perceived, responded to, and internalized ACEs. For example, experiences of psychological abuse or neglect may have been normalized or underreported—particularly among men—due to gendered social norms that emphasize emotional restraint and self-control (Stoltenborgh et al., 2013). This cultural backdrop may partly explain why psychological abuse was associated with a lower risk of mortality in both sexes in our study. In addition, elevated child mortality among those who experienced severe ACEs during wartime and the immediate postwar period may have resulted in survivor bias, whereby only individuals who were resilient to such adversities survived to be included in the older adult sample. These cultural and historical factors are essential to consider when interpreting both the strengths and limitations of our study.

### Limitations

Our study has several limitations. First, although this was a large-scale, multi-municipality study, the sample was not nationally representative. In separate analysis using the same cohort, nonrespondents were found to be older, shorter of lower socioeconomic status, and less socially engaged than respondents (Tani et al., 2020). Therefore, individuals at higher risk of mortality may have been underrepresented, potentially leading to an underestimation of the association between ACEs and mortality. Second, we restricted our analysis to older adults who did not use long-term care insurance, as a proxy for being physically and cognitively independent. However, it is possible that some individuals with physical or cognitive decline who did not utilize long-term care services were inadvertently included. Third, ACEs were assessed retrospectively via self-report questionnaires, which may introduce recall bias (Baldwin et al., 2019). In our study, for example, parental loss was associated with a higher risk of mortality in women but recall accuracy over several decades remains uncertain. Fourth, the ACE items were designed more than 65 years after the end of the Pacific War and may not fully reflect the contextual realities of participants’ childhood experiences prior to age 18. Fifth, we categorized the cumulative number of ACEs into three groups: 0, 1, and 2 or more. This simplification may have obscured subtle dose–response relationships, which should be considered when interpreting the findings. However, this decision was made to avoid small cell sizes and unstable estimates, as the number of participants with three or more ACEs was limited. Analyses using more granular classifications (e.g., 3 + or 4 + ACEs) revealed no substantial differences in the results, supporting the robustness of our categorization (results not shown). Finally, as stated in the Methods section, we employed modified Poisson regression with robust error variance because the proportional hazards assumption required for Cox regression was not satisfied in our data. This method required the observation period to be equal across participants. As a result, some individuals who died between the two waves (2013–2019) but after the predefined observation period may have been excluded from the analysis. This may have underestimated the association between ACEs and mortality.

## Conclusions

This study demonstrated that specific ACEs components and the cumulative number of ACEs were associated with an increased risk of mortality in later life, even among older adults without physical or cognitive decline. These findings underscore the enduring impact of early-life adversity and highlight the importance of adopting a life-course approach in public health and geriatric care. Screening for ACEs in older populations may aid in identifying individuals at elevated risk of premature mortality and support the development of targeted interventions. Expanding trauma-informed care frameworks to include aging populations and addressing gender-specific and socioeconomic disparities—particularly among women who experienced parental loss—may help mitigate the long-term consequences of childhood adversity. Further research is warranted to elucidate the underlying mechanisms of these associations and to inform the design of effective, tailored prevention strategies.

## Supplementary Information

Below is the link to the electronic supplementary material.

Supplementary file1 (PPTX 45 KB)

Supplementary file2 (PPTX 50 KB)

Supplementary file3 (PPTX 50 KB)

Supplementary file4 (PPTX 52 KB)

Supplementary file5 (PPTX 49 KB)

## Data Availability

The data that support the findings of this study are available from the Japan Gerontological Evaluation Study (JAGES), but restrictions apply to the availability of these data, which were used under license for the current study, and so are not publicly available. Data are available from the authors upon reasonable request and with permission of JAGES.

## References

[CR1] Amemiya, A., Fujiwara, T., Murayama, H., Tani, Y., & Kondo, K. (2018). Adverse childhood experiences and higher-level functional limitations among older Japanese people: Results from the JAGES study. *The Journals of Gerontology. Series a, Biological Sciences and Medical Sciences,**73*(2), 261–266. 10.1093/gerona/glx097 28525611 10.1093/gerona/glx097

[CR2] Amemiya, A., Fujiwara, T., Shirai, K., Kondo, K., Oksanen, T., Pentti, J., & Vahtera, J. (2019). Association between adverse childhood experiences and adult diseases in older adults: A comparative cross-sectional study in Japan and Finland. *British Medical Journal Open,**9*(8), e024609. 10.1136/bmjopen-2018-024609 10.1136/bmjopen-2018-024609 PMC6720330 31446402

[CR3] Anda, R. F., Whitfield, C. L., Felitti, V. J., Chapman, D., Edwards, V. J., Dube, S. R., & Williamson, D. F. (2002). Adverse childhood experiences, alcoholic parents, and later risk of alcoholism and depression. *Psychiatric Services,**53*(8), 1001–1009. 10.1176/appi.ps.53.8.1001 12161676 10.1176/appi.ps.53.8.1001

[CR4] Anonymized for Review #1

[CR5] Anonymized for Review #2

[CR6] Anonymized for Review #3

[CR7] Ashida, T., Fujiwara, T., & Kondo, K. (2023). Association between adverse childhood experiences and social integration among older people in Japan: Results from the JAGES study. *Archives of Gerontology and Geriatrics,**114*, 105099. 10.1016/j.archger.2023.105099 37329767 10.1016/j.archger.2023.105099

[CR8] Baldwin, J. R., Reuben, A., Newbury, J. B., & Danese, A. (2019). Agreement between prospective and retrospective measures of childhood maltreatment: A systematic review and meta-analysis. *JAMA Psychiatry,**76*(6), 584–593. 10.1001/jamapsychiatry.2019.0097 30892562 10.1001/jamapsychiatry.2019.0097 PMC6551848

[CR9] Bale, T. L., & Epperson, C. N. (2015). Sex differences and stress across the lifespan. *Nature Neuroscience,**18*(10), 1413–1420. 10.1038/nn.4112 26404716 10.1038/nn.4112 PMC4620712

[CR10] Bellis, M. A., Hughes, K., Leckenby, N., Hardcastle, K. A., Perkins, C., & Lowey, H. (2015). Measuring mortality and the burden of adult disease associated with adverse childhood experiences in England: A national survey. *Journal of Public Health,**37*(3), 445–454. 10.1093/pubmed/fdu065 25174044 10.1093/pubmed/fdu065 PMC4552010

[CR11] Bente Kjær, L., Claus Høstrup, V., Dorte, R., Mogens, V., Trine, M.-O., & Bodil Hammer, B. (2018). Attendance of routine childcare visits in primary care for children of mothers with depression: A nationwide population-based cohort study. *British Journal of General Practice,**68*(667), e97. 10.3399/bjgp18X694565 10.3399/bjgp18X694565 PMC5774969 29335326

[CR12] Brown, D. W., Anda, R. F., Tiemeier, H., Felitti, V. J., Edwards, V. J., Croft, J. B., & Giles, W. H. (2009). Adverse childhood experiences and the risk of premature mortality. *American Journal of Preventive Medicine,**37*(5), 389–396. 19840693 10.1016/j.amepre.2009.06.021

[CR13] Brown, D. W., Anda, R. F., Felitti, V. J., Edwards, V. J., Malarcher, A. M., Croft, J. B., & Giles, W. H. (2010). Adverse childhood experiences are associated with the risk of lung cancer: A prospective cohort study. *BMC Public Health,**10*, 20. 10.1186/1471-2458-10-20 20085623 10.1186/1471-2458-10-20 PMC2826284

[CR14] Cavanagh, S. E., Crissey, S. R., & Raley, R. K. (2008). Family structure history and adolescent romance. *Journal of Marriage and Family,**70*(3), 698–714. 10.1111/j.1741-3737.2008.00515.x

[CR15] Chen, Y.-H., Chiou, H.-Y., Tang, C.-H., & Lin, H.-C. (2010). Risk of death by unnatural causes during early childhood in offspring of parents with mental illness. *The American Journal of Psychiatry,**167*(2), 198–205. 10.1176/appi.ajp.2009.09070979 19952076 10.1176/appi.ajp.2009.09070979

[CR16] Chen, E., Turiano, N. A., Mroczek, D. K., & Miller, G. E. (2016). Association of reports of childhood abuse and all-cause mortality rates in women. *JAMA Psychiatry,**73*(9), 920–927. 10.1001/jamapsychiatry.2016.1786 27540997 10.1001/jamapsychiatry.2016.1786 PMC5234580

[CR17] Cheong, E. V., Carol, S., Darren, D., & Patricia, M. K. (2017). Adverse childhood experiences (ACEs) and later-life depression: Perceived social support as a potential protective factor. *British Medical Journal Open,**7*(9), e013228. 10.1136/bmjopen-2016-013228 10.1136/bmjopen-2016-013228 PMC5588961 28864684

[CR18] D’Arcy-Bewick, S., Turiano, N., Sutin, A. R., Terracciano, A., & O’Súilleabháin, P. S. (2023). Adverse childhood experiences and all-cause mortality risk in adulthood. *Child Abuse & Neglect,**144*, 106386. 10.1016/j.chiabu.2023.106386 37542995 10.1016/j.chiabu.2023.106386

[CR19] Davidsen, K. A., Christiansen, E., Haubek, D., Asmussen, J., Ranning, A., Thorup, A. A. E., Nordentoft, M., Harder, S., & Bilenberg, N. (2021). Parental mental illness, attendance at preventive child healthcare and dental caries in the offspring: A nation-wide population-based cohort study. *Social Psychiatry and Psychiatric Epidemiology,**56*(4), 583–592. 10.1007/s00127-020-01936-3 32812086 10.1007/s00127-020-01936-3

[CR20] de Souza, A. F., Máximo, Rd. O., de Oliveira, D. C., Ramírez, P. C., Luiz, M. M., Delinocente, M. L. B., Santos, J. L. F., Steptoe, A., de Oliveira, C., & Alexandre, Td. S. (2022). Gender differences in the association between adverse events in childhood or adolescence and the risk of premature mortality. *Scientific Reports,**12*(1), 19118. 10.1038/s41598-022-23443-y 36352182 10.1038/s41598-022-23443-y PMC9646814

[CR21] Dong, M., Giles, W. H., Felitti, V. J., Dube, S. R., Williams, J. E., Chapman, D. P., & Anda, R. F. (2004). Insights into causal pathways for ischemic heart disease: Adverse childhood experiences study. *Circulation,**110*(13), 1761–1766. 10.1161/01.Cir.0000143074.54995.7f 15381652 10.1161/01.CIR.0000143074.54995.7F

[CR22] Dye, H. L. (2020). Is emotional abuse as harmful as physical and/or sexual abuse? *Journal of Child & Adolescent Trauma,**13*(4), 399–407. 10.1007/s40653-019-00292-y 33269040 10.1007/s40653-019-00292-y PMC7683637

[CR23] Evans, S. E., Steel, A. L., & DiLillo, D. (2013). Child maltreatment severity and adult trauma symptoms: Does perceived social support play a buffering role? *Child Abuse & Neglect,**37*(11), 934–943. 10.1016/j.chiabu.2013.03.005 23623620 10.1016/j.chiabu.2013.03.005 PMC3758446

[CR24] Felitti, V. J., Anda, R. F., Nordenberg, D., Williamson, D. F., Spitz, A. M., Edwards, V., Koss, M. P., & Marks, J. S. (1998). Relationship of childhood abuse and household dysfunction to many of the leading causes of death in adults: The Adverse Childhood Experiences (ACE) study. *American Journal of Preventive Medicine,**14*(4), 245–258. 9635069 10.1016/s0749-3797(98)00017-8

[CR25] Fujiwara, T., Kondo, K., Shirai, K., Suzuki, K., & Kawachi, I. (2014). Associations of childhood socioeconomic status and adulthood height with functional limitations among Japanese older people: Results from the JAGES 2010 Project. *Journals of Gerontology. Series a, Biological Sciences and Medical Sciences,**69*(7), 852–859. 10.1093/gerona/glt189 24285745 10.1093/gerona/glt189

[CR26] Fujiwara, T. (2022). Impact of adverse childhood experience on physical and mental health: A life-course epidemiology perspective. *Psychiatry and Clinical Neurosciences*, *n/a*(n/a). 10.1111/pcn.13464 10.1111/pcn.13464 36002401

[CR27] Giordano, P. C., Longmore, M. A., & Manning, W. D. (2006). Gender and the meanings of adolescent romantic relationships: A focus on boys. *American Sociological Review,**71*(2), 260–287. 10.1177/000312240607100205

[CR28] Guldin, M.-B., Li, J., Pedersen, H. S., Obel, C., Agerbo, E., Gissler, M., Cnattingius, S., Olsen, J., & Vestergaard, M. (2015). Incidence of suicide among persons who had a parent who died during their childhood: A population-based cohort study. *JAMA Psychiatry,**72*(12), 1227–1234. 10.1001/jamapsychiatry.2015.2094 26558351 10.1001/jamapsychiatry.2015.2094

[CR29] Hailey Maier, E., & Lachman, M. E. (2000). Consequences of early parental loss and separation for health and well-being in midlife. *International Journal of Behavioral Development,**24*(2), 183–189. 10.1080/016502500383304

[CR30] Heuckendorff, S., Johansen, M. N., Johnsen, S. P., Overgaard, C., & Fonager, K. (2021). Parental mental health conditions and use of healthcare services in children the first year of life– A register-based, nationwide study. *BMC Public Health,**21*(1), 557. 10.1186/s12889-021-10625-y 33743653 10.1186/s12889-021-10625-y PMC7981963

[CR31] Hiyoshi, A., Berg, L., Grotta, A., Almquist, Y., & Rostila, M. (2021). Parental death in childhood and pathways to increased mortality across the life course in Stockholm, Sweden: A cohort study. *PLoS Medicine,**18*(3), e1003549. 10.1371/journal.pmed.1003549 33705393 10.1371/journal.pmed.1003549 PMC7951838

[CR32] Johnson, J., Chaudieu, I., Ritchie, K., Scali, J., Ancelin, M. L., & Ryan, J. (2020). The extent to which childhood adversity and recent stress influence all-cause mortality risk in older adults. *Psychoneuroendocrinology,**111*, 104492. 10.1016/j.psyneuen.2019.104492 31704635 10.1016/j.psyneuen.2019.104492

[CR33] Kelly-Irving, M., Lepage, B., Dedieu, D., Bartley, M., Blane, D., Grosclaude, P., Lang, T., & Delpierre, C. (2013). Adverse childhood experiences and premature all-cause mortality. *European Journal of Epidemiology,**28*(9), 721–734. 10.1007/s10654-013-9832-9 23887883 10.1007/s10654-013-9832-9 PMC3787798

[CR34] Kessler, R. C., McLaughlin, K. A., Green, J. G., Gruber, M. J., Sampson, N. A., Zaslavsky, A. M., Aguilar-Gaxiola, S., Alhamzawi, A. O., Alonso, J., Angermeyer, M., Benjet, C., Bromet, E., Chatterji, S., de Girolamo, G., Demyttenaere, K., Fayyad, J., Florescu, S., Gal, G., Gureje, O., … Williams, D. R. (2010). Childhood adversities and adult psychopathology in the WHO world mental health surveys. *British Journal of Psychiatry,**197*(5), 378–385. 10.1192/bjp.bp.110.080499 10.1192/bjp.bp.110.080499 PMC2966503 21037215

[CR35] Larson, K., & Halfon, N. (2013). Parental divorce and adult longevity. *International Journal of Public Health,**58*(1), 89–97. 10.1007/s00038-012-0373-x 22674375 10.1007/s00038-012-0373-x

[CR36] Lebra, T. S. (1976). *Japanese patterns of behavior*. University of Hawaii Press. https://ci.nii.ac.jp/ncid/BA04328370

[CR37] Lee, C., & Ryff, C. D. (2019). Pathways linking combinations of early-life adversities to adult mortality: Tales that vary by gender. *Social Science & Medicine*. 10.1016/j.socscimed.2019.112566 31585378 10.1016/j.socscimed.2019.112566 PMC6894498

[CR38] Lee, J., Tan, C. S., & Chia, K. S. (2009). A practical guide for multivariate analysis of dichotomous outcomes. *Annals of the Academy of Medicine, Singapore,**38*(8), 714–719. 19736577

[CR39] Lyngsøe, B. K., Rytter, D., Munk-Olsen, T., Vestergaard, C. H., Christensen, K. S., & Bech, B. H. (2019). Maternal depression and primary healthcare use for children: A population-based cohort study in Denmark. *British Journal of General Practice,**69*(680), e182–e189. 10.3399/bjgp18X700733 10.3399/bjgp18X700733 PMC6400607 30559112

[CR40] Manning, C., & Gregoire, A. (2009). Effects of parental mental illness on children. *Psychiatry,**8*(1), 7–9. 10.1016/j.mppsy.2008.10.012

[CR41] Martin, L. R., Friedman, H. S., Clark, K. M., & Tucker, J. S. (2005). Longevity following the experience of parental divorce. *Social Science & Medicine,**61*(10), 2177–2189. 15936133 10.1016/j.socscimed.2005.04.027

[CR42] Matsuyama, Y., Fujiwara, T., Aida, J., Watt, R. G., Kondo, N., Yamamoto, T., Kondo, K., & Osaka, K. (2016). Experience of childhood abuse and later number of remaining teeth in older Japanese: A life-course study from Japan Gerontological Evaluation Study project. *Community Dentistry and Oral Epidemiology,**44*(6), 531–539. 27417875 10.1111/cdoe.12246

[CR43] McKay, S. (1998). The effects of armed conflict on girls and women. *Peace and Conflict: Journal of Peace Psychology,**4*(4), 381–392. 10.1207/s15327949pac0404_6

[CR44] McNutt, L. A., Wu, C., Xue, X., & Hafner, J. P. (2003). Estimating the relative risk in cohort studies and clinical trials of common outcomes. *American Journal of Epidemiology,**157*(10), 940–943. 10.1093/aje/kwg074 12746247 10.1093/aje/kwg074

[CR45] Merrick, M. T., Ford, D. C., Ports, K. A., Guinn, A. S., Chen, J., Klevens, J., Metzler, M., Jones, C. M., Simon, T. R., Daniel, V. M., Ottley, P., & Mercy, J. A. (2019). Vital signs: Estimated proportion of adult health problems attributable to adverse childhood experiences and implications for prevention - 25 states, 2015–2017. *MMWR. Morbidity and Mortality Weekly Report,**68*(44), 999–1005. 10.15585/mmwr.mm6844e1 31697656 10.15585/mmwr.mm6844e1 PMC6837472

[CR46] Ministry of Health, L. a. W. (2021). *Number of deaths by age group* https://honkawa2.sakura.ne.jp/1557.html

[CR47] Moore, A. W., & Piel, L. H. (n.d.). *The wartime evacuation of children, 1944–1945*. Manchester University. Retrieved 17/10 from https://sites.manchester.ac.uk/japanese-childhood/home/home-en/topics-2/evacuee-children/

[CR48] Peter, C. (n.d.). *Children, education and war, 1931–1945*. The University of Manchester. Retrieved 17/10 from https://sites.manchester.ac.uk/japanese-childhood/home/home-en/topics-2/children-education-and-war-1931-1945/

[CR49] Piel, L. H. (2016). Japanese adolescents and the wartime labor service, 1941–45: Service or exploitation? *Japanese Studies,**36*(3), 361–381. 10.1080/10371397.2016.1253001

[CR50] Remes, H., Martikainen, P., & Valkonen, T. (2011). The effects of family type on child mortality. *European Journal of Public Health,**21*(6), 688–693. 10.1093/eurpub/ckq159 21051470 10.1093/eurpub/ckq159

[CR51] Renneberg, C. K., Brund, R. B. K., Heuckendorff, S., Bech, B. H., & Fonager, K. (2023). Children of parents with different severities of mental health conditions have higher risk of somatic morbidity: A Danish nationwide register-based cohort study. *BMC Public Health,**23*(1), 810. 10.1186/s12889-023-15714-8 37138276 10.1186/s12889-023-15714-8 PMC10155386

[CR52] Rogers, N. T., Power, C., & Pinto Pereira, S. M. (2021). Child maltreatment, early life socioeconomic disadvantage and all-cause mortality in mid-adulthood: Findings from a prospective British birth cohort. *British Medical Journal Open,**11*(9), e050914. 10.1136/bmjopen-2021-050914 10.1136/bmjopen-2021-050914 PMC8461284 34551950

[CR53] Rubin, D. B. (1996). Multiple imputation after 18+ years. *Journal of the American Statistical Association,**91*(434), 473–489. 10.2307/2291635

[CR54] Sheikh, M. A. (2018). Psychological abuse, substance abuse distress, dissatisfaction with friendships, and incident psychiatric problems. *Journal of Psychosomatic Research,**108*, 78–84. 10.1016/j.jpsychores.2018.03.001 29602329 10.1016/j.jpsychores.2018.03.001

[CR55] Sheikh, M. A. (2021). Confounding, mediation, or independent effect? Childhood psychological abuse, mental health, mood/psychological state, COPD, and migraine. *Journal of Interpersonal Violence,**36*(15/16), NP8706–NP8723. 10.1177/0886260519844773 31046532 10.1177/0886260519844773

[CR56] Shera, L., & Sher, L. (2017). Parental alienation: The impact on men’s mental health. *International Journal of Adolescent Medicine and HealTh,**29*(3), 1–5. 10.1515/ijamh-2015-0083 26565536 10.1515/ijamh-2015-0083

[CR57] Silventoinen, K. (2003). Determinants of variation in adult body height. *Journal of Biosocial Science,**35*(2), 263–285. 10.1017/s0021932003002633 12664962 10.1017/s0021932003002633

[CR58] Smith, K. R., Hanson, H. A., Norton, M. C., Hollingshaus, M. S., & Mineau, G. P. (2014). Survival of offspring who experience early parental death: Early life conditions and later-life mortality. *Social Science & Medicine,**119*, 180–190. 24530028 10.1016/j.socscimed.2013.11.054 PMC4087105

[CR59] Stoltenborgh, M., Bakermans-Kranenburg, M. J., van Ijzendoorn, M. H., & Alink, L. R. (2013). Cultural-geographical differences in the occurrence of child physical abuse? A meta-analysis of global prevalence. *International Journal of Psychology,**48*(2), 81–94. 10.1080/00207594.2012.697165 23597008 10.1080/00207594.2012.697165

[CR60] Tamanoi, M. A. (2020). The Origins and Plight of Sensō Koji (War Orphans) in Postwar Japan. *Asia Pacific Journal: Japan Focus*, *18*(13). https://apjjf.org/2020/13/tamanoi

[CR61] Tani, Y., Fujiwara, T., & Kondo, K. (2020). Association between adverse childhood experiences and dementia in older Japanese adults. *JAMA Network Open,**3*(2), e1920740–e1920740. 10.1001/jamanetworkopen.2019.20740 32031646 10.1001/jamanetworkopen.2019.20740

[CR62] Tani, Y., Fujiwara, T., & Kondo, K. (2021). Adverse childhood experiences and dementia: Interactions with social capital in the Japan gerontological evaluation study cohort. *American Journal of Preventive Medicine,**61*(2), 225–234. 10.1016/j.amepre.2021.01.045 33985835 10.1016/j.amepre.2021.01.045

[CR63] Under-Secretary-General, P. (2003). Women suffer disproportionately during and after war. security council told during day-long debate on women, peace and security| Meetings Coverage and Press Releases, United Nations

[CR64] Waehrer, G. M., Miller, T. R., Silverio Marques, S. C., Oh, D. L., & Burke Harris, N. (2020). Disease burden of adverse childhood experiences across 14 states. *PLoS ONE,**15*(1), e0226134. 10.1371/journal.pone.0226134 31990910 10.1371/journal.pone.0226134 PMC6986706

[CR65] Wang, Y.-X., Sun, Y., Missmer, S. A., Rexrode, K. M., Roberts, A. L., Chavarro, J. E., & Rich-Edwards, J. W. (2023a). Association of early life physical and sexual abuse with premature mortality among female nurses: Prospective cohort study. *BMJ,**381*, e073613. 10.1136/bmj-2022-073613 37137504 10.1136/bmj-2022-073613 PMC10155244

[CR66] Wang, Y., Chen, X., Zhou, K., & Zhang, H. (2023b). A meta-analysis of the effects of childhood maltreatment on elderly depression [article]. *Trauma, Violence & Abuse,**24*(3), 1593–1607. 10.1177/15248380211073838 10.1177/15248380211073838 35232293

[CR67] Yesavage, J. A., Brink, T. L., Rose, T. L., Lum, O., Huang, V., Adey, M., & Leirer, V. O. (1982). Development and validation of a geriatric depression screening scale: A preliminary report. *Journal of Psychiatric Research,**17*(1), 37–49. 10.1016/0022-3956(82)90033-4 7183759 10.1016/0022-3956(82)90033-4

[CR68] Yu, J., Patel, R. A., Haynie, D. L., Vidal-Ribas, P., Govender, T., Sundaram, R., & Gilman, S. E. (2022). Adverse childhood experiences and premature mortality through mid-adulthood: A five-decade prospective study. *The Lancet Regional Health - Americas,**15*, 100349. 36467261 10.1016/j.lana.2022.100349 PMC9718480

[CR69] Zhang, J., & Yu, K. F. (1998). What’s the relative risk? A method of correcting the odds ratio in cohort studies of common outcomes. *JAMA,**280*(19), 1690–1691. 10.1001/jama.280.19.1690 9832001 10.1001/jama.280.19.1690

