# Supplementary material for: Adverse Childhood Experiences and Mortality at Old Age: A Longitudinal Study from the Japan Gerontological Evaluation Study
Source: J Child Adolesc Trauma. 2025 Dec 20;19(1):259–72. doi: 10.1007/s40653-025-00732-y (PMC13004767; doi:10.1007/s40653-025-00732-y)
Supplement: Supplementary file 1 — Supplementary file1 (PPTX 45 KB) [file 40653_2025_732_MOESM1_ESM.pptx]

## Slide 1
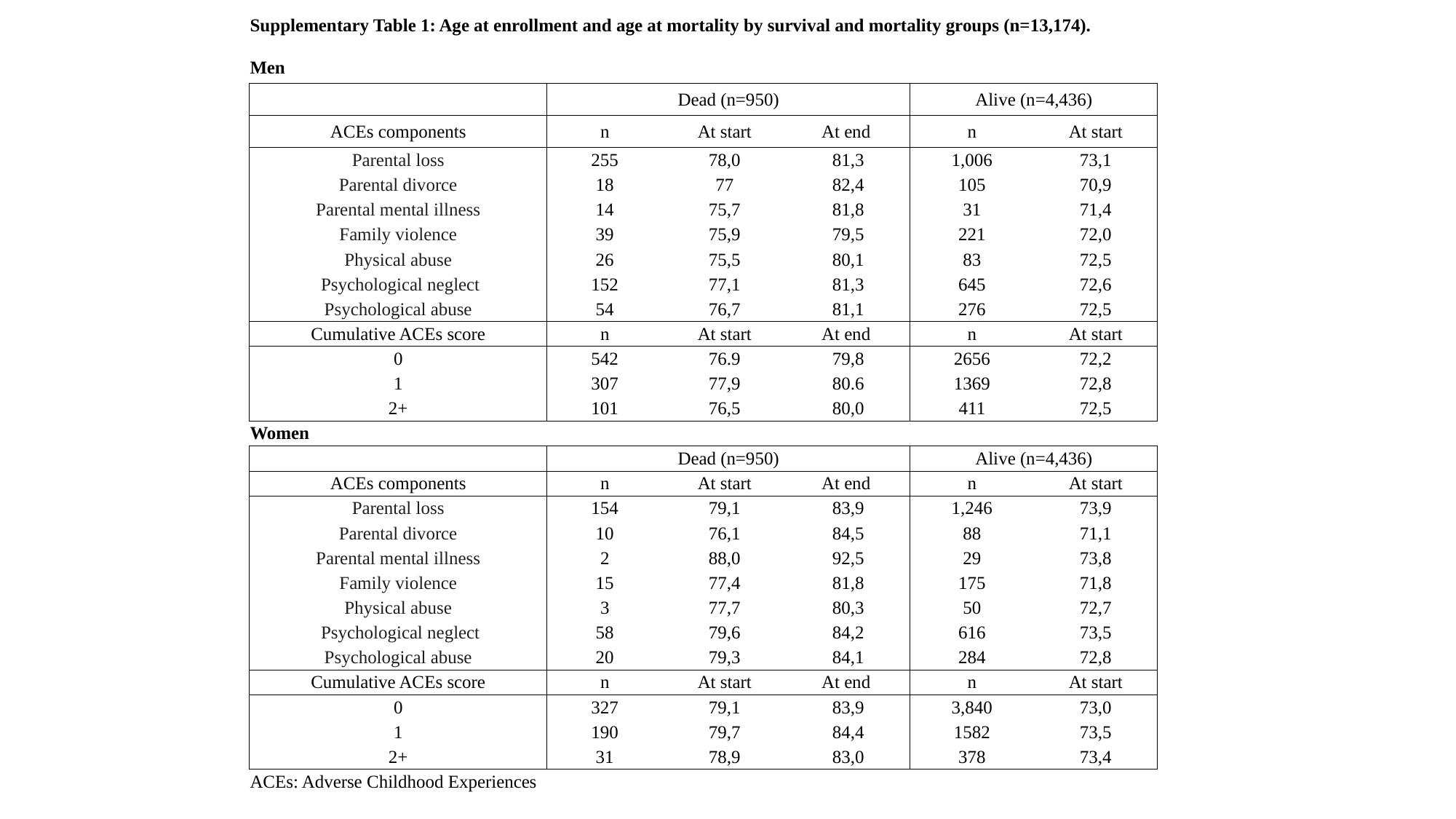

| Supplementary Table 1: Age at enrollment and age at mortality by survival and mortality groups (n=13,174). | | | | | | |
| --- | --- | --- | --- | --- | --- | --- |
| Men | | | | | | |
| | | Dead (n=950) | | | Alive (n=4,436) | |
| ACEs components | | n | At start | At end | n | At start |
| Parental loss | | 255 | 78,0 | 81,3 | 1,006 | 73,1 |
| Parental divorce | | 18 | 77 | 82,4 | 105 | 70,9 |
| Parental mental illness | | 14 | 75,7 | 81,8 | 31 | 71,4 |
| Family violence | | 39 | 75,9 | 79,5 | 221 | 72,0 |
| Physical abuse | | 26 | 75,5 | 80,1 | 83 | 72,5 |
| Psychological neglect | | 152 | 77,1 | 81,3 | 645 | 72,6 |
| Psychological abuse | | 54 | 76,7 | 81,1 | 276 | 72,5 |
| Cumulative ACEs score | | n | At start | At end | n | At start |
| 0 | | 542 | 76.9 | 79,8 | 2656 | 72,2 |
| 1 | | 307 | 77,9 | 80.6 | 1369 | 72,8 |
| 2+ | | 101 | 76,5 | 80,0 | 411 | 72,5 |
| Women | | | | | | |
| | | Dead (n=950) | | | Alive (n=4,436) | |
| ACEs components | | n | At start | At end | n | At start |
| Parental loss | | 154 | 79,1 | 83,9 | 1,246 | 73,9 |
| Parental divorce | | 10 | 76,1 | 84,5 | 88 | 71,1 |
| Parental mental illness | | 2 | 88,0 | 92,5 | 29 | 73,8 |
| Family violence | | 15 | 77,4 | 81,8 | 175 | 71,8 |
| Physical abuse | | 3 | 77,7 | 80,3 | 50 | 72,7 |
| Psychological neglect | | 58 | 79,6 | 84,2 | 616 | 73,5 |
| Psychological abuse | | 20 | 79,3 | 84,1 | 284 | 72,8 |
| Cumulative ACEs score | | n | At start | At end | n | At start |
| 0 | | 327 | 79,1 | 83,9 | 3,840 | 73,0 |
| 1 | | 190 | 79,7 | 84,4 | 1582 | 73,5 |
| 2+ | | 31 | 78,9 | 83,0 | 378 | 73,4 |
| ACEs: Adverse Childhood Experiences | | | | | | |
| | | | | | | |
