# Supplementary material for: Adverse Childhood Experiences and Mortality at Old Age: A Longitudinal Study from the Japan Gerontological Evaluation Study
Source: J Child Adolesc Trauma. 2025 Dec 20;19(1):259–72. doi: 10.1007/s40653-025-00732-y (PMC13004767; doi:10.1007/s40653-025-00732-y)
Supplement: Supplementary file 2 — Supplementary file2 (PPTX 50 KB) [file 40653_2025_732_MOESM2_ESM.pptx]

## Slide 1
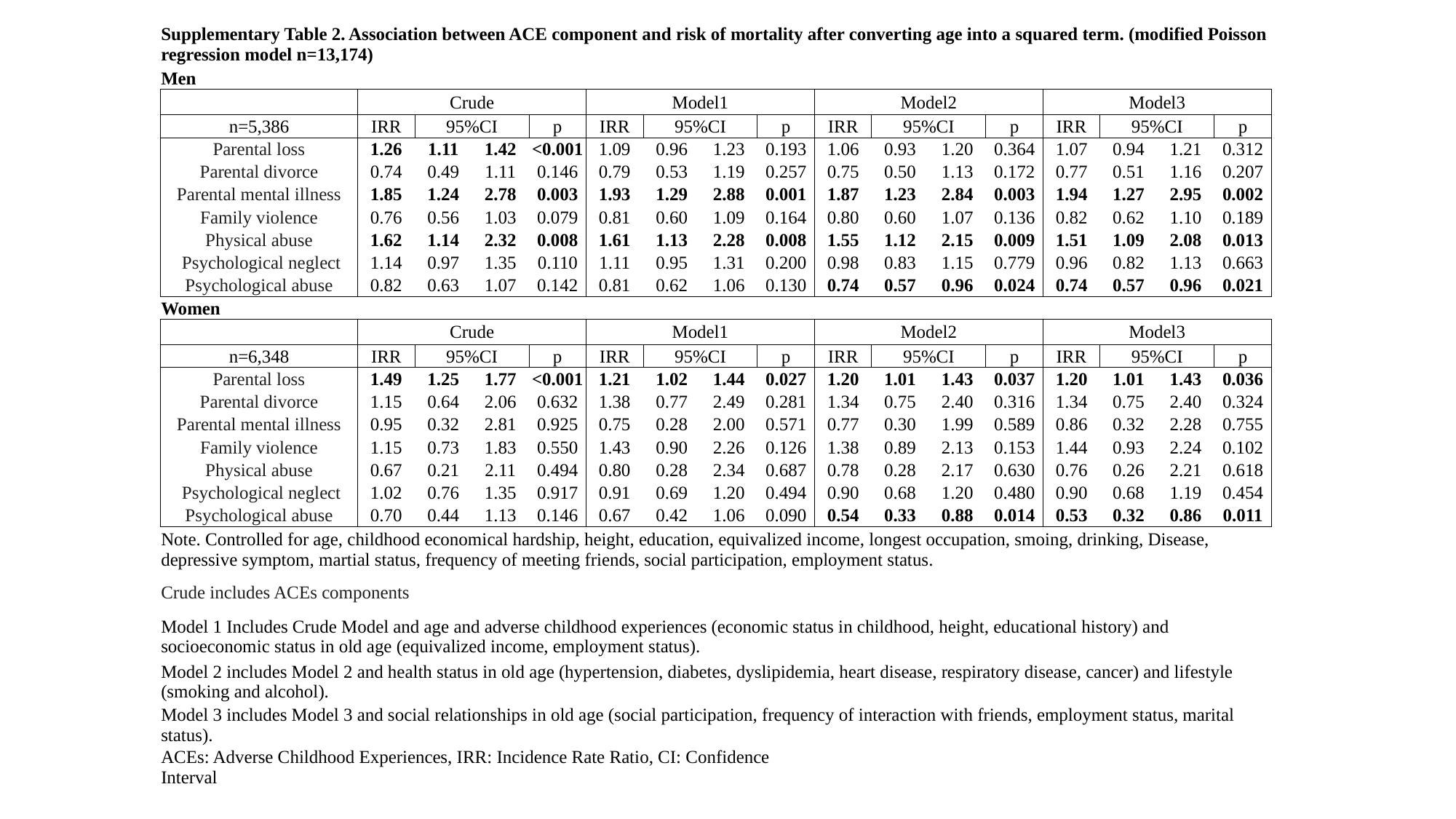

| Supplementary Table 2. Association between ACE component and risk of mortality after converting age into a squared term. (modified Poisson regression model n=13,174) | | | | | | | | | | | | | | | | | |
| --- | --- | --- | --- | --- | --- | --- | --- | --- | --- | --- | --- | --- | --- | --- | --- | --- | --- |
| Men | | | | | | | | | | | | | | | | | |
| | | Crude | | | | Model1 | | | | Model2 | | | | Model3 | | | |
| n=5,386 | | IRR | 95%CI | | p | IRR | 95%CI | | p | IRR | 95%CI | | p | IRR | 95%CI | | p |
| Parental loss | | 1.26 | 1.11 | 1.42 | <0.001 | 1.09 | 0.96 | 1.23 | 0.193 | 1.06 | 0.93 | 1.20 | 0.364 | 1.07 | 0.94 | 1.21 | 0.312 |
| Parental divorce | | 0.74 | 0.49 | 1.11 | 0.146 | 0.79 | 0.53 | 1.19 | 0.257 | 0.75 | 0.50 | 1.13 | 0.172 | 0.77 | 0.51 | 1.16 | 0.207 |
| Parental mental illness | | 1.85 | 1.24 | 2.78 | 0.003 | 1.93 | 1.29 | 2.88 | 0.001 | 1.87 | 1.23 | 2.84 | 0.003 | 1.94 | 1.27 | 2.95 | 0.002 |
| Family violence | | 0.76 | 0.56 | 1.03 | 0.079 | 0.81 | 0.60 | 1.09 | 0.164 | 0.80 | 0.60 | 1.07 | 0.136 | 0.82 | 0.62 | 1.10 | 0.189 |
| Physical abuse | | 1.62 | 1.14 | 2.32 | 0.008 | 1.61 | 1.13 | 2.28 | 0.008 | 1.55 | 1.12 | 2.15 | 0.009 | 1.51 | 1.09 | 2.08 | 0.013 |
| Psychological neglect | | 1.14 | 0.97 | 1.35 | 0.110 | 1.11 | 0.95 | 1.31 | 0.200 | 0.98 | 0.83 | 1.15 | 0.779 | 0.96 | 0.82 | 1.13 | 0.663 |
| Psychological abuse | | 0.82 | 0.63 | 1.07 | 0.142 | 0.81 | 0.62 | 1.06 | 0.130 | 0.74 | 0.57 | 0.96 | 0.024 | 0.74 | 0.57 | 0.96 | 0.021 |
| Women | | | | | | | | | | | | | | | | | |
| | | Crude | | | | Model1 | | | | Model2 | | | | Model3 | | | |
| n=6,348 | | IRR | 95%CI | | p | IRR | 95%CI | | p | IRR | 95%CI | | p | IRR | 95%CI | | p |
| Parental loss | | 1.49 | 1.25 | 1.77 | <0.001 | 1.21 | 1.02 | 1.44 | 0.027 | 1.20 | 1.01 | 1.43 | 0.037 | 1.20 | 1.01 | 1.43 | 0.036 |
| Parental divorce | | 1.15 | 0.64 | 2.06 | 0.632 | 1.38 | 0.77 | 2.49 | 0.281 | 1.34 | 0.75 | 2.40 | 0.316 | 1.34 | 0.75 | 2.40 | 0.324 |
| Parental mental illness | | 0.95 | 0.32 | 2.81 | 0.925 | 0.75 | 0.28 | 2.00 | 0.571 | 0.77 | 0.30 | 1.99 | 0.589 | 0.86 | 0.32 | 2.28 | 0.755 |
| Family violence | | 1.15 | 0.73 | 1.83 | 0.550 | 1.43 | 0.90 | 2.26 | 0.126 | 1.38 | 0.89 | 2.13 | 0.153 | 1.44 | 0.93 | 2.24 | 0.102 |
| Physical abuse | | 0.67 | 0.21 | 2.11 | 0.494 | 0.80 | 0.28 | 2.34 | 0.687 | 0.78 | 0.28 | 2.17 | 0.630 | 0.76 | 0.26 | 2.21 | 0.618 |
| Psychological neglect | | 1.02 | 0.76 | 1.35 | 0.917 | 0.91 | 0.69 | 1.20 | 0.494 | 0.90 | 0.68 | 1.20 | 0.480 | 0.90 | 0.68 | 1.19 | 0.454 |
| Psychological abuse | | 0.70 | 0.44 | 1.13 | 0.146 | 0.67 | 0.42 | 1.06 | 0.090 | 0.54 | 0.33 | 0.88 | 0.014 | 0.53 | 0.32 | 0.86 | 0.011 |
| Note. Controlled for age, childhood economical hardship, height, education, equivalized income, longest occupation, smoing, drinking, Disease, depressive symptom, martial status, frequency of meeting friends, social participation, employment status. | | | | | | | | | | | | | | | | | |
| Crude includes ACEs components | | | | | | | | | | | | | | | | | |
| Model 1 Includes Crude Model and age and adverse childhood experiences (economic status in childhood, height, educational history) and socioeconomic status in old age (equivalized income, employment status). | | | | | | | | | | | | | | | | | |
| Model 2 includes Model 2 and health status in old age (hypertension, diabetes, dyslipidemia, heart disease, respiratory disease, cancer) and lifestyle (smoking and alcohol). | | | | | | | | | | | | | | | | | |
| Model 3 includes Model 3 and social relationships in old age (social participation, frequency of interaction with friends, employment status, marital status). | | | | | | | | | | | | | | | | | |
| ACEs: Adverse Childhood Experiences, IRR: Incidence Rate Ratio, CI: Confidence Interval | | | | | | | | | | | | | | | | | |
