# Supplementary material for: Adverse Childhood Experiences and Mortality at Old Age: A Longitudinal Study from the Japan Gerontological Evaluation Study
Source: J Child Adolesc Trauma. 2025 Dec 20;19(1):259–72. doi: 10.1007/s40653-025-00732-y (PMC13004767; doi:10.1007/s40653-025-00732-y)
Supplement: Supplementary file 3 — Supplementary file3 (PPTX 50 KB) [file 40653_2025_732_MOESM3_ESM.pptx]

## Slide 1
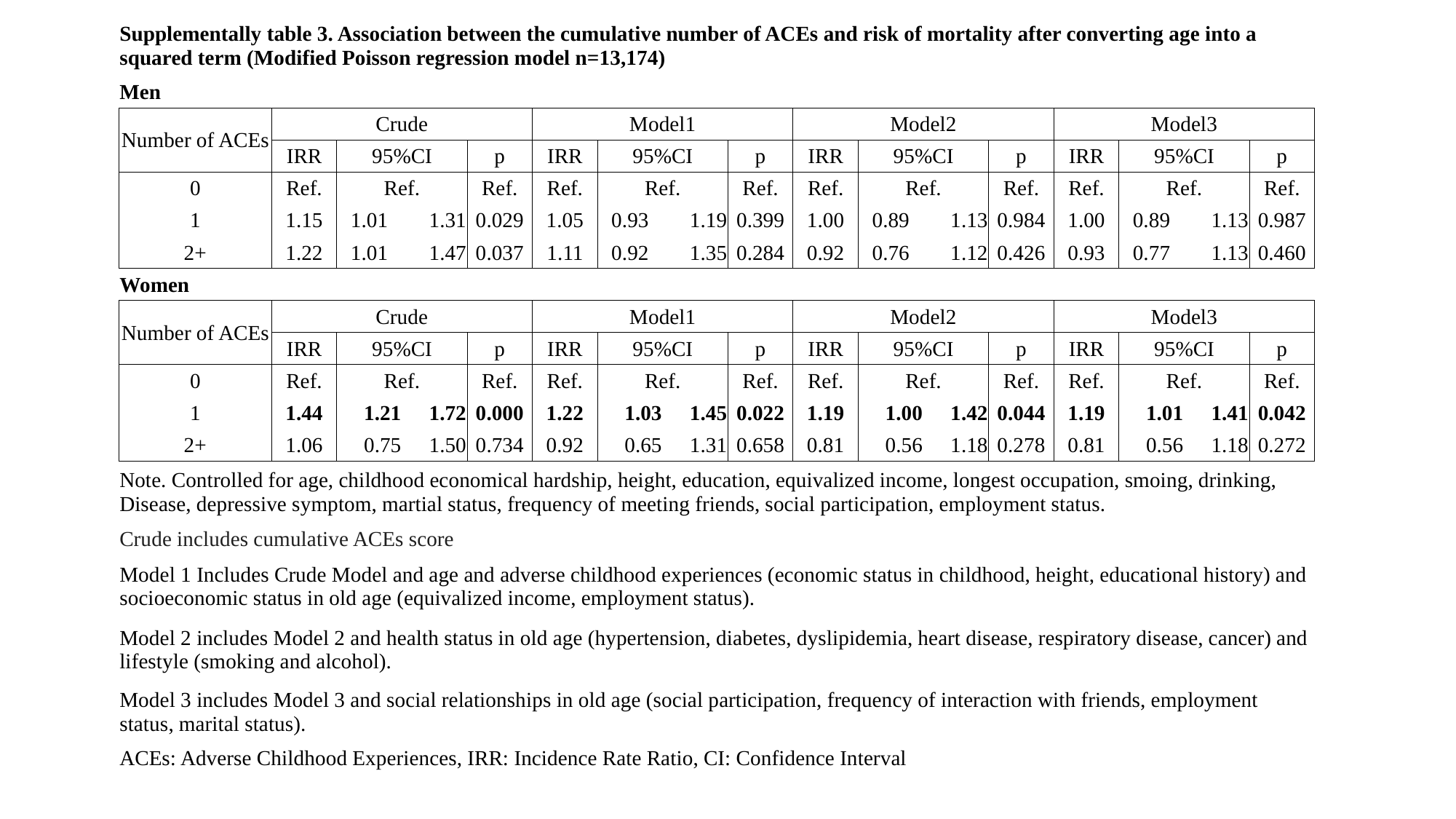

| Supplementally table 3. Association between the cumulative number of ACEs and risk of mortality after converting age into a squared term (Modified Poisson regression model n=13,174) | | | | | | | | | | | | | | | | |
| --- | --- | --- | --- | --- | --- | --- | --- | --- | --- | --- | --- | --- | --- | --- | --- | --- |
| Men | | | | | | | | | | | | | | | | |
| Number of ACEs | Crude | | | | Model1 | | | | Model2 | | | | Model3 | | | |
| | IRR | 95%CI | | p | IRR | 95%CI | | p | IRR | 95%CI | | p | IRR | 95%CI | | p |
| 0 | Ref. | Ref. | | Ref. | Ref. | Ref. | | Ref. | Ref. | Ref. | | Ref. | Ref. | Ref. | | Ref. |
| 1 | 1.15 | 1.01 | 1.31 | 0.029 | 1.05 | 0.93 | 1.19 | 0.399 | 1.00 | 0.89 | 1.13 | 0.984 | 1.00 | 0.89 | 1.13 | 0.987 |
| 2+ | 1.22 | 1.01 | 1.47 | 0.037 | 1.11 | 0.92 | 1.35 | 0.284 | 0.92 | 0.76 | 1.12 | 0.426 | 0.93 | 0.77 | 1.13 | 0.460 |
| Women | | | | | | | | | | | | | | | | |
| Number of ACEs | Crude | | | | Model1 | | | | Model2 | | | | Model3 | | | |
| | IRR | 95%CI | | p | IRR | 95%CI | | p | IRR | 95%CI | | p | IRR | 95%CI | | p |
| 0 | Ref. | Ref. | | Ref. | Ref. | Ref. | | Ref. | Ref. | Ref. | | Ref. | Ref. | Ref. | | Ref. |
| 1 | 1.44 | 1.21 | 1.72 | 0.000 | 1.22 | 1.03 | 1.45 | 0.022 | 1.19 | 1.00 | 1.42 | 0.044 | 1.19 | 1.01 | 1.41 | 0.042 |
| 2+ | 1.06 | 0.75 | 1.50 | 0.734 | 0.92 | 0.65 | 1.31 | 0.658 | 0.81 | 0.56 | 1.18 | 0.278 | 0.81 | 0.56 | 1.18 | 0.272 |
| Note. Controlled for age, childhood economical hardship, height, education, equivalized income, longest occupation, smoing, drinking, Disease, depressive symptom, martial status, frequency of meeting friends, social participation, employment status. | | | | | | | | | | | | | | | | |
| Crude includes cumulative ACEs score | | | | | | | | | | | | | | | | |
| Model 1 Includes Crude Model and age and adverse childhood experiences (economic status in childhood, height, educational history) and socioeconomic status in old age (equivalized income, employment status). | | | | | | | | | | | | | | | | |
| Model 2 includes Model 2 and health status in old age (hypertension, diabetes, dyslipidemia, heart disease, respiratory disease, cancer) and lifestyle (smoking and alcohol). | | | | | | | | | | | | | | | | |
| Model 3 includes Model 3 and social relationships in old age (social participation, frequency of interaction with friends, employment status, marital status). | | | | | | | | | | | | | | | | |
| ACEs: Adverse Childhood Experiences, IRR: Incidence Rate Ratio, CI: Confidence Interval | | | | | | | | | | | | | | | | |
