# Supplementary material for: Adverse Childhood Experiences and Mortality at Old Age: A Longitudinal Study from the Japan Gerontological Evaluation Study
Source: J Child Adolesc Trauma. 2025 Dec 20;19(1):259–72. doi: 10.1007/s40653-025-00732-y (PMC13004767; doi:10.1007/s40653-025-00732-y)
Supplement: Supplementary file 4 — Supplementary file4 (PPTX 52 KB) [file 40653_2025_732_MOESM4_ESM.pptx]

## Slide 1
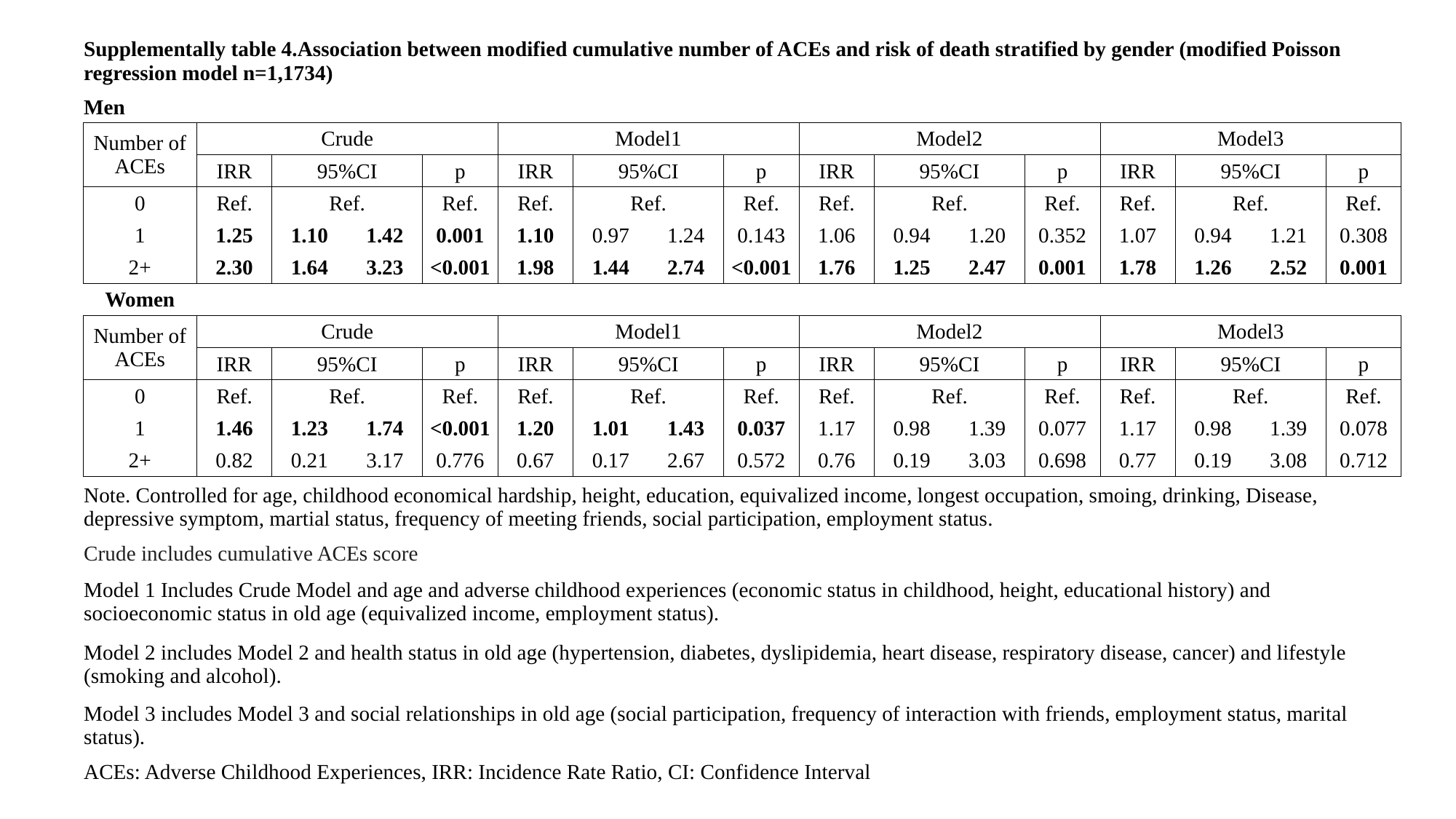

| Supplementally table 4.Association between modified cumulative number of ACEs and risk of death stratified by gender (modified Poisson regression model n=1,1734) | | | | | | | | | | | | | | | | |
| --- | --- | --- | --- | --- | --- | --- | --- | --- | --- | --- | --- | --- | --- | --- | --- | --- |
| Men | | | | | | | | | | | | | | | | |
| Number of ACEs | Crude | | | | Model1 | | | | Model2 | | | | Model3 | | | |
| | IRR | 95%CI | | p | IRR | 95%CI | | p | IRR | 95%CI | | p | IRR | 95%CI | | p |
| 0 | Ref. | Ref. | | Ref. | Ref. | Ref. | | Ref. | Ref. | Ref. | | Ref. | Ref. | Ref. | | Ref. |
| 1 | 1.25 | 1.10 | 1.42 | 0.001 | 1.10 | 0.97 | 1.24 | 0.143 | 1.06 | 0.94 | 1.20 | 0.352 | 1.07 | 0.94 | 1.21 | 0.308 |
| 2+ | 2.30 | 1.64 | 3.23 | <0.001 | 1.98 | 1.44 | 2.74 | <0.001 | 1.76 | 1.25 | 2.47 | 0.001 | 1.78 | 1.26 | 2.52 | 0.001 |
| Women | | | | | | | | | | | | | | | | |
| Number of ACEs | Crude | | | | Model1 | | | | Model2 | | | | Model3 | | | |
| | IRR | 95%CI | | p | IRR | 95%CI | | p | IRR | 95%CI | | p | IRR | 95%CI | | p |
| 0 | Ref. | Ref. | | Ref. | Ref. | Ref. | | Ref. | Ref. | Ref. | | Ref. | Ref. | Ref. | | Ref. |
| 1 | 1.46 | 1.23 | 1.74 | <0.001 | 1.20 | 1.01 | 1.43 | 0.037 | 1.17 | 0.98 | 1.39 | 0.077 | 1.17 | 0.98 | 1.39 | 0.078 |
| 2+ | 0.82 | 0.21 | 3.17 | 0.776 | 0.67 | 0.17 | 2.67 | 0.572 | 0.76 | 0.19 | 3.03 | 0.698 | 0.77 | 0.19 | 3.08 | 0.712 |
| Note. Controlled for age, childhood economical hardship, height, education, equivalized income, longest occupation, smoing, drinking, Disease, depressive symptom, martial status, frequency of meeting friends, social participation, employment status. | | | | | | | | | | | | | | | | |
| Crude includes cumulative ACEs score | | | | | | | | | | | | | | | | |
| Model 1 Includes Crude Model and age and adverse childhood experiences (economic status in childhood, height, educational history) and socioeconomic status in old age (equivalized income, employment status). | | | | | | | | | | | | | | | | |
| Model 2 includes Model 2 and health status in old age (hypertension, diabetes, dyslipidemia, heart disease, respiratory disease, cancer) and lifestyle (smoking and alcohol). | | | | | | | | | | | | | | | | |
| Model 3 includes Model 3 and social relationships in old age (social participation, frequency of interaction with friends, employment status, marital status). | | | | | | | | | | | | | | | | |
| ACEs: Adverse Childhood Experiences, IRR: Incidence Rate Ratio, CI: Confidence Interval | | | | | | | | | | | | | | | | |
#
