# Supplementary material for: Adverse Childhood Experiences and Mortality at Old Age: A Longitudinal Study from the Japan Gerontological Evaluation Study
Source: J Child Adolesc Trauma. 2025 Dec 20;19(1):259–72. doi: 10.1007/s40653-025-00732-y (PMC13004767; doi:10.1007/s40653-025-00732-y)
Supplement: Supplementary file 5 — Supplementary file5 (PPTX 49 KB) [file 40653_2025_732_MOESM5_ESM.pptx]

## Slide 1
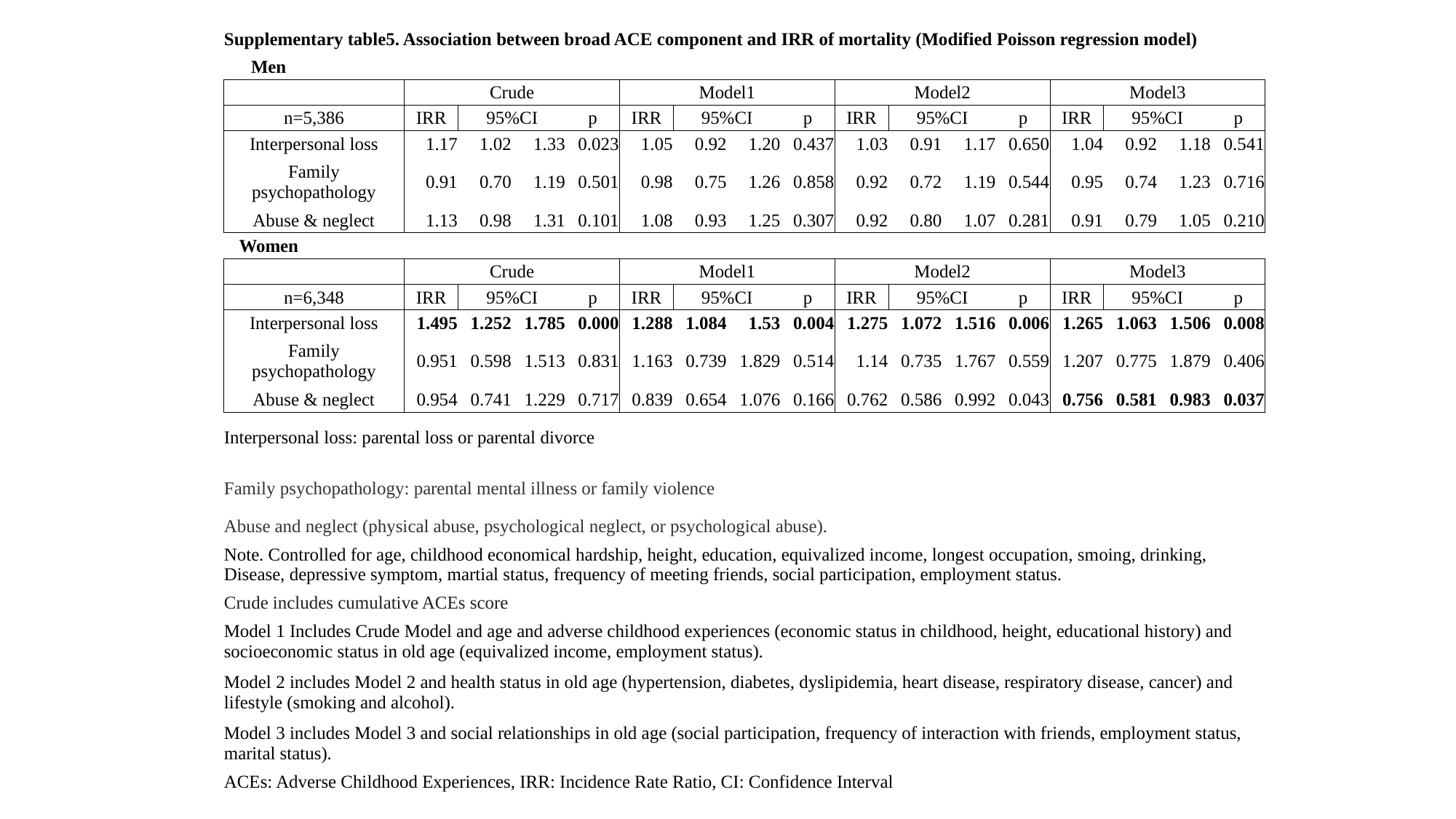

| Supplementary table5. Association between broad ACE component and IRR of mortality (Modified Poisson regression model) | | | | | | | | | | | | | | | | | |
| --- | --- | --- | --- | --- | --- | --- | --- | --- | --- | --- | --- | --- | --- | --- | --- | --- | --- |
| Men | | | | | | | | | | | | | | | | | |
| | | Crude | | | | Model1 | | | | Model2 | | | | Model3 | | | |
| n=5,386 | | IRR | 95%CI | | p | IRR | 95%CI | | p | IRR | 95%CI | | p | IRR | 95%CI | | p |
| Interpersonal loss | | 1.17 | 1.02 | 1.33 | 0.023 | 1.05 | 0.92 | 1.20 | 0.437 | 1.03 | 0.91 | 1.17 | 0.650 | 1.04 | 0.92 | 1.18 | 0.541 |
| Family psychopathology | | 0.91 | 0.70 | 1.19 | 0.501 | 0.98 | 0.75 | 1.26 | 0.858 | 0.92 | 0.72 | 1.19 | 0.544 | 0.95 | 0.74 | 1.23 | 0.716 |
| Abuse & neglect | | 1.13 | 0.98 | 1.31 | 0.101 | 1.08 | 0.93 | 1.25 | 0.307 | 0.92 | 0.80 | 1.07 | 0.281 | 0.91 | 0.79 | 1.05 | 0.210 |
| Women | | | | | | | | | | | | | | | | | |
| | | Crude | | | | Model1 | | | | Model2 | | | | Model3 | | | |
| n=6,348 | | IRR | 95%CI | | p | IRR | 95%CI | | p | IRR | 95%CI | | p | IRR | 95%CI | | p |
| Interpersonal loss | | 1.495 | 1.252 | 1.785 | 0.000 | 1.288 | 1.084 | 1.53 | 0.004 | 1.275 | 1.072 | 1.516 | 0.006 | 1.265 | 1.063 | 1.506 | 0.008 |
| Family psychopathology | | 0.951 | 0.598 | 1.513 | 0.831 | 1.163 | 0.739 | 1.829 | 0.514 | 1.14 | 0.735 | 1.767 | 0.559 | 1.207 | 0.775 | 1.879 | 0.406 |
| Abuse & neglect | | 0.954 | 0.741 | 1.229 | 0.717 | 0.839 | 0.654 | 1.076 | 0.166 | 0.762 | 0.586 | 0.992 | 0.043 | 0.756 | 0.581 | 0.983 | 0.037 |
| Interpersonal loss: parental loss or parental divorce | | | | | | | | | | | | | | | | | |
| Family psychopathology: parental mental illness or family violence | | | | | | | | | | | | | | | | | |
| Abuse and neglect (physical abuse, psychological neglect, or psychological abuse). | | | | | | | | | | | | | | | | | |
| Note. Controlled for age, childhood economical hardship, height, education, equivalized income, longest occupation, smoing, drinking, Disease, depressive symptom, martial status, frequency of meeting friends, social participation, employment status. | | | | | | | | | | | | | | | | | |
| Crude includes cumulative ACEs score | | | | | | | | | | | | | | | | | |
| Model 1 Includes Crude Model and age and adverse childhood experiences (economic status in childhood, height, educational history) and socioeconomic status in old age (equivalized income, employment status). | | | | | | | | | | | | | | | | | |
| Model 2 includes Model 2 and health status in old age (hypertension, diabetes, dyslipidemia, heart disease, respiratory disease, cancer) and lifestyle (smoking and alcohol). | | | | | | | | | | | | | | | | | |
| Model 3 includes Model 3 and social relationships in old age (social participation, frequency of interaction with friends, employment status, marital status). | | | | | | | | | | | | | | | | | |
| ACEs: Adverse Childhood Experiences, IRR: Incidence Rate Ratio, CI: Confidence Interval | | | | | | | | | | | | | | | | | |
